# Redox and structural determinants of mitochondrial complex III inhibition by triphenylphosphonium-conjugated atovaquone analogs

**DOI:** 10.1016/j.redox.2026.104370

**Published:** 2026-08-28

**Authors:** Bruna Rafaela Pereira Resende, Gang Cheng, Gabriel Canard, Didier Siri, Vanessa Leone, Micael Hardy, Balaraman Kalyanaraman

**Affiliations:** a Aix-Marseille Univ, CNRS, ICR, UMR 7273, Marseille, France; b Department of Biophysics, 8701 Watertown Plank Road, Milwaukee, WI, United States; c Aix-Marseille Univ, CNRS, CINaM, UMR 7325, Marseille, France

**Keywords:** Atovaquone, Mitochondrial complex III, Superoxide, Redox-cycling, Cancer

## Abstract

Growing evidence indicates that cancer cell mitochondria remain functional and represent attractive therapeutic targets. Mitochondria-targeted drug delivery commonly uses triphenylphosphonium (TPP^+^)-conjugated compounds, which preferentially accumulate in cancer cell mitochondria because of their highly negative membrane potential. Many TPP^+^-conjugated agents inhibit mitochondrial electron transport chain complexes I and II, suppressing mitochondrial respiration and cancer cell proliferation. Recent studies suggest that electron-withdrawing substituents, such as trifluoromethyl groups, on the TPP^+^ phenyl rings alter electron density around the phosphorus center, enhancing mitochondrial uncoupling activity and antiproliferative effects. To determine how TPP^+^ electronic substituents influence redox properties, mitochondrial complex III interactions, and biological activity, we used mitochondria-targeted atovaquone (**Mito-ATO**) as a model system.

Atovaquone (**ATO**), a hydroxy-1,4-naphthoquinone and the only FDA-approved mitochondrial complex III inhibitor, is currently undergoing clinical evaluation for cancer therapy. We synthesized a series of mitochondria-targeted ATO derivatives **(MitoR-ATOs**) containing electron-donating or electron-withdrawing substituents on the TPP^+^ moiety. Their effects on enzymatic superoxide generation were assessed by EPR spin trapping, mitochondrial oxygen consumption by Seahorse XF96 analysis, complex III Qi-site binding by computational modeling, and cancer cell proliferation using IncuCyte live-cell imaging. Unexpectedly, several **MitoR-ATO** derivatives emerged as potent complex III inhibitors through enhanced binding at the Qi site.

Structure–activity relationship analysis revealed that the intrinsic redox properties of TPP^+^-conjugated MitoR-ATO analogs alone do not predict biological activity. Instead, accurate prediction of the therapeutic efficacy of mitochondria-targeted complex III inhibitors requires integrating redox properties with computational analyses of ligand–protein interactions at the mitochondrial complex III Qi site.

## Highlights

• Electronic effects on TPP ^+^ modulate Mito-ATO redox properties.
• Redox changes did not significantly alter cancer cell growth inhibition.
• Mito-ATO derivatives target the mitochondrial complex III Qi site.
• Alkyl linker elongation abolished complex III inhibition and activity.

## Graphical abstract

**Figure:**
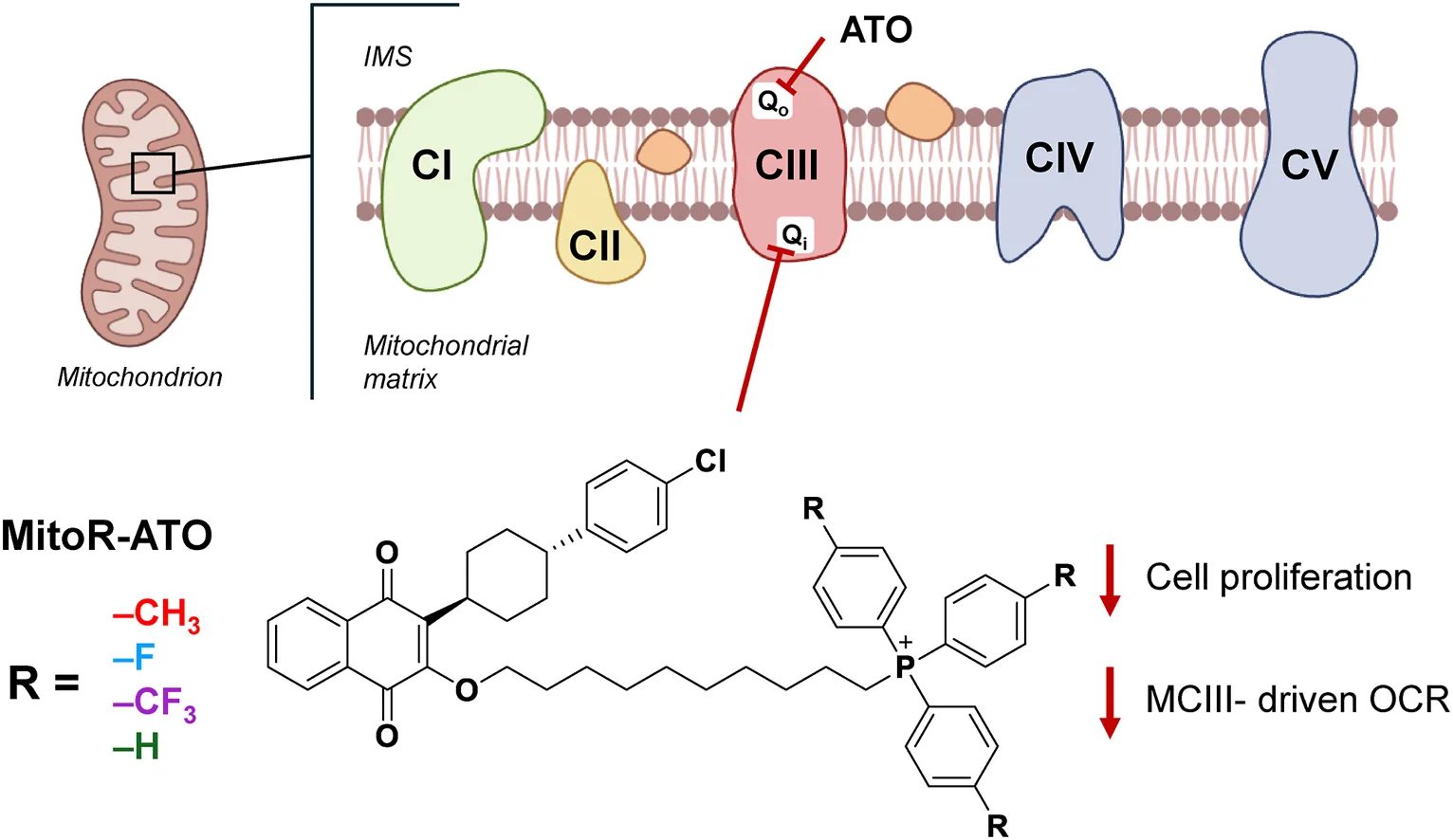

## 1 Introduction

Cancer cells preferentially rely on glycolysis for energy production even under oxygen-replete conditions, a metabolic adaptation known as the Warburg effect [1,2]. This phenotype was initially attributed to defective mitochondria that forced cancer cells to depend on glycolysis. However, accumulating evidence from our laboratory and others has established that mitochondria in cancer cells remain functional and represent attractive therapeutic targets for cancer prevention and treatment, including immunotherapy [[3], [4], [5], [6], [7], [8], [9]]. Targeted delivery of drugs to mitochondria commonly uses delocalized lipophilic cations, such as the triphenylphosphonium (TPP^+^) moiety, which accumulate more readily in cancer cell mitochondria due to their higher negative membrane potential [10,11]. Numerous TPP^+^-conjugated compounds, including **Mito-Met**, **Mito-Q**, and redox-crippled **Mito-Q**, disrupt mitochondrial bioenergetics by inhibiting the electron transport chain, thereby suppressing oxidative phosphorylation and cancer cell proliferation [12,13].

Recent studies have shown that introducing electron-withdrawing substituents, such as trifluoromethyl (–CF_3_), into the phenyl rings of TPP^+^ alters the electronic environment surrounding the phosphorus atom [14,15]. These modifications were associated with enhanced mitochondrial uncoupling in C2C12 cells and increased antitumor efficacy of Mito-Met in pancreatic cancer models [16]. Based on these reports, we hypothesized that systematic modulation of the electronic properties of the TPP^+^ moiety would proportionally influence redox behavior, reactive oxygen species (ROS) generation, mitochondrial complex III inhibition, and ultimately antiproliferative activity. Unexpectedly, our findings did not support this simple relationship. Instead, they revealed that alterations in redox properties alone do not reliably predict biological activity, indicating that additional structural determinants govern the interactions of mitochondria-targeted compounds with their molecular targets.

Because quinone-containing compounds undergo redox cycling, we further hypothesized that electron-donating and electron-withdrawing substituents on the TPP^+^ moiety would modulate the redox cycling and superoxide-generating properties of mitochondria-targeted quinones. Atovaquone (**ATO**), a hydroxy-1,4-naphthoquinone, was originally developed as an antimalarial agent through inhibition of the cytochrome *bc*_*1*_ complex in *Plasmodium falciparum* [17,18]. **ATO** is currently the only FDA-approved antimalarial drug that targets mitochondrial complex III and has recently been repurposed as a potential anticancer agent [19]. To enhance its therapeutic efficacy, we previously developed TPP^+^-conjugated **ATO** derivatives (**MitoR-ATO**), which exhibited nearly 100-fold greater antiproliferative activity than ATO in breast cancer cells [20].

**Fig. 1:**
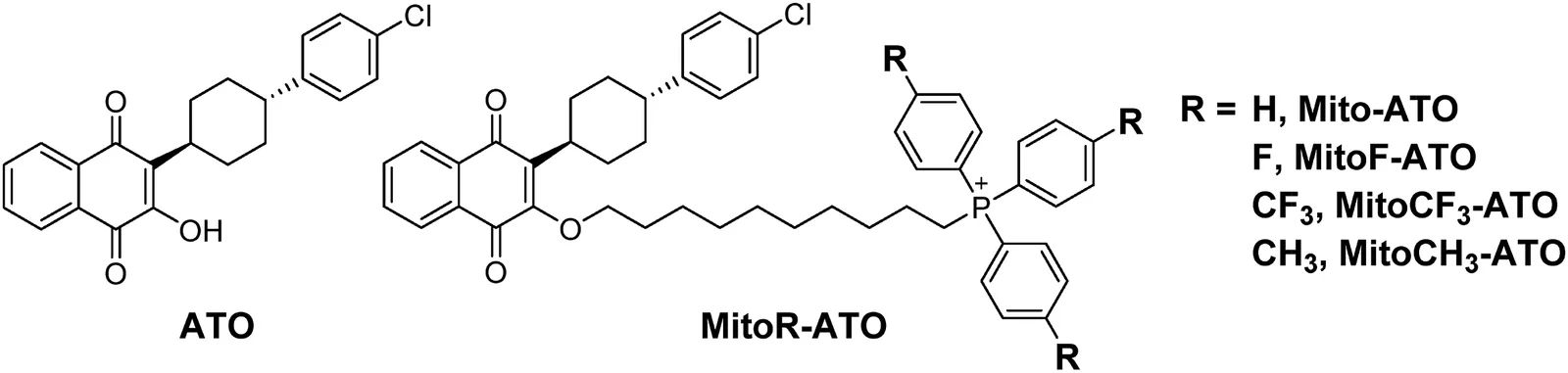
Molecular structures of ATO and Mito-ATO with electron-withdrawing and electron-donating substituents on TPP^+^ (MitoR-ATO).

In the present study, we synthesized a new series of **MitoR-ATO** derivatives containing electron-donating or electron-withdrawing substituents on the TPP^+^ moiety (Fig. 1). We systematically evaluated how these electronic modifications influence redox cycling, ROS generation, mitochondrial respiration, and cancer cell proliferation. To further define their mechanism of action, we performed molecular docking simulations to characterize the interactions of **ATO** and its mitochondria-targeted analogs with mitochondrial complex III. These studies revealed unexpected differences in ligand binding, including altered interactions at the complex III Qᵢ site, providing a structural explanation for the observed biological responses. Together, these findings establish that redox modulation alone is insufficient to predict the biological activity of mitochondria-targeted quinones and provide a mechanistic framework for the rational design of next-generation mitochondrial therapeutics for cancer, radiosensitization, malaria, and other diseases involving mitochondrial dysfunction.

## 2 Materials and methods

### 2.1 Synthesis

**Fig. 2:**
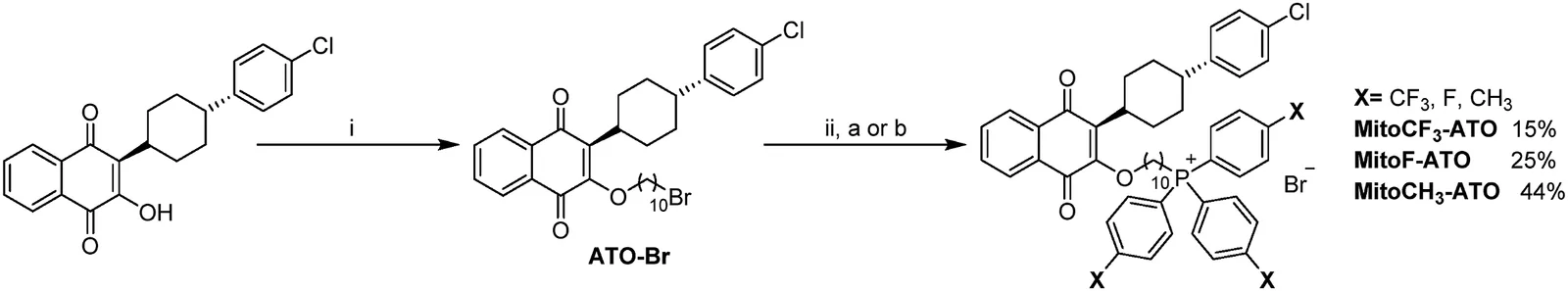
**Synthesis of MitoR-ATO. Reagents and** conditions: i, Br-(CH_2_)_10_-Br, K_2_CO_3_, DMF, 70 °C, 24h, (68%); ii.a, (4-FC_6_H_4_)_3_P, or (4-CF_3_C_6_H_4_)_3_P, neat, 90 °C, 16-288h; ii.b, (4-CH_3_C_6_H_4_)_3_P, CH_3_CN, reflux, 16h.

The mitochondria-targeted analogs of atovaquone (**MitoR-ATO**) were prepared in two steps by reacting dibromodecane with atovaquone in the presence of potassium carbonate in dry N,N-Dimethylformamide (DMF). **MitoCF_3_-ATO**, **MitoF-ATO,** and **MitoCH_3_-ATO** were synthesized by adding the corresponding triphenylphosphine analog to the bromoalkylated atovaquone **ATO-Br** (Fig. 2).

### 2.2 General

All chemicals and organic solvents were commercially available and used without further purification. 5-Diisopropyloxy-phosphoryl-5-methyl-1-pyrroline-N-oxide (DIPPMPO) was synthesized as previously described [21]. Reaction progress was monitored by thin-layer chromatography on Merck silica gel ^60^F_254_ plates.

^1^H and ^13^C NMR spectra were acquired on a Bruker DPX AVANCE 400 spectrometer (400.13 MHz for ^1^H; 75.54 MHz for ^13^C) equipped with a quattro nucleus probe. Spectra were recorded in deuterated chloroform (CDCl_3_) using CDCl_3_ and tetramethylsilane as internal references. Chemical shifts (*δ*) are reported in ppm and coupling constants (J) in Hz.

Electron paramagnetic resonance (EPR) spectra were obtained at room temperature using a Bruker ELEXSYS spectrometer operating at 9.5 GHz with 100 kHz field modulation. Spectrophotometric measurements were performed using a TECAN Infinite M-Nano plus system.

### 2.3 MitoCH_3_-ATO

In an ice-cold bath, potassium carbonate (0.34 g, 2.45 mmol) was added to a mixture of **ATO** (1.0 g, 2.73 mmol) and 1,10-dibromodecane (1.81 g, 8.18 mmol) in dry DMF (12 mL) under an inert atmosphere. The mixture was then stirred at 70 °C for 24 h. The crude product was recovered in diethyl ether (Et_2_O; 60 mL) and washed with water (H_2_O; 2 × 10 mL). The organic layer was dried over sodium sulfate (Na_2_SO_4_), and the solvent was removed under reduced pressure. Purification by flash chromatography on silica gel (pentane/Et_2_O from 100% to 7% Et_2_O) yielded the corresponding **ATO-Br** as a yellow solid (1.86 g, 68% yield).

^1^H NMR (400.13 MHz, CDCl_3_), *δ* 8.08-8.01 (m, 2H), 7.73-7.66 (m, 2H), 7.29-7.26 (m, 2H), 7.20-7.17 (m, 2H), 4.33 (t, *J* = 6.6 Hz, 2H), 3.39 (t, *J* = 6.8 Hz, 2H), 3.22 (tt, *J* = 12.0, 3.3 Hz, 1H), 2.62 (tt, *J* = 12.0, 3.3 Hz, 1H), 2.19 (qd, *J* = 12.8, 3.3 Hz, 2H), 1.98 (dd, *J* = 13.5, 3.0 Hz, 2H), 1.88-1.80 (m, 4H), 1.74 (dd, *J* = 13.5, 3.0 Hz, 2H), 1.60-1.48 (m, 4H), 1.43-1.32 (m, 10H).

In a pear-shaped flask under an inert atmosphere, ATO-Br (0.3 g, 0.52 mmol) and tri(*p*-tolyl)-phosphine (0.62 g, 2.05 mmol) in acetonitrile (CH_3_CN; 2 mL) were stirred at 80 °C for 16 h. Crude is concentrated under reduced pressure. Purification by flash chromatography on silica gel (Et_2_O 100% to dichloromethane [CH_2_Cl_2_] 100%, then CH_2_Cl_2_/ethanol [EtOH] from 0% to 10% EtOH) yielded the corresponding **MitoCH_3_-ATO** as a golden-brown solid (0.204 g, 44% yield).

HR-MS calculated for **MitoCH_3_-ATO** C_53_H_59_ClO_3_P^+^ [M]^+^: 809.3885; found: 809.3884.

^1^H NMR (400.13 MHz, CDCl_3_), *δ* 8.07-8.01 (m, 2H), 7.70-7.65 (m, 8H), 7.48-7.45 (m, 6H), 7.26-7.24 (m, 2H), 7.18-7.16 (m, 2H), 4.3 (t, *J =* 6.5 Hz, 2H), 3.66-3.59 (m, 2H), 3.20 (tt, *J =* 12.2, 3.2 Hz, 1H), 2.61 (tt, *J =* 12.2, 3.2 Hz, 1H), 2.47 (s, 9H), 2.17 (qd, *J =* 12.6, 3.1 Hz, 2H), 1.96 (dd, *J =* 13.3, 2.8 Hz, 2H), 1.81 (quint, *J =* 6.7 Hz, 2H), 1.72 (dd, *J =* 13.2, 2.8 Hz, 2H), 1.55-1.43 (m, 6H), 1.35-1.26 (m, 10H).

^31^P NMR (161.98 MHz, CDCl_3_), *δ* 23.3.

^13^C NMR (100.58 MHz, CDCl_3_), *δ* 187.8, 158.1, 146.0, 138.6, 133.8, 133.6 (d, ^2^*J*_*C-P*_ = 10.3 Hz), 133.1, 131.1 (d, ^3^*J*_*C-P*_ = 12.5 Hz), 128.4, 128.2, 126.3, 125.9, 115.4 (d, ^*1*^*J*_*C-P*_ = 88.1 Hz), 74.1, 43.3, 35.3, 34.5, 30.4, 29.9, 29.6, 29.4, 29.3, 29.3, 25.9, 22.8, 21.8.

### 2.4 MitoF-ATO

In a pear-shaped flask under an inert atmosphere, **ATO-Br** (0.3 g, 0.52 mmol) and tris(4-fluorophenyl)phosphine (0.65 g, 2.05 mmol) were stirred at 80 °C for 16 h. Crude was recovered in CH_2_Cl_2_, dropwise added to Et_2_O (250 mL), and left to precipitate overnight at −20 °C. Et_2_O was removed, and the crude was concentrated under reduced pressure. Purification by flash chromatography on silica gel (Et_2_O 100% to CH_2_Cl_2_ 100%, then CH_2_Cl_2_/EtOH from 0% to 10% EtOH) yielded the corresponding **MitoF-ATO** as a golden solid (0.116 g, 25% yield).

HR-MS calculated for **MitoF-ATO** C_50_H_50_ClF_3_O_3_P^+^ [M]^+^ 821.3133, found 821.3127.

^1^H NMR (400.13 MHz, CDCl_3_), *δ* 8.07-8.05 (m, 1H), 8.02-8.00 (m, 1H), 7.97-7.90 (m, 6H), 7.73-7.65 (m, 2H), 7.44-7.38 (m, 6H), 7.26-7.23 (m, 2H), 7.19-7.16 (m, 2H), 4.3 (t, *J =* 6.7 Hz, 2H), 4.00-3.93 (m, 2H), 3.20 (tt, *J =* 12.3, 3.6 Hz, 1H), 2.61 (tt, *J =* 12.2, 3.6 Hz, 1H), 2.17 (qd, *J =* 12.8, 3.5 Hz, 2H), 1.96 (dd, *J =* 13.4, 3.5 Hz, 2H), 1.82 (quint, *J =* 7.1 Hz, 2H), 1.73 (dd, *J =* 13.3, 2.8 Hz, 2H), 1.59-1.43 (m, 8H), 1.37-1.29 (m, 8H).

^19^F NMR (376.95 MHz, CDCl_3_), *δ* −99.3.

^31^P NMR (161.98 MHz, CDCl_3_), *δ* 24.1.

^13^C NMR (100.58 MHz, CDCl_3_), *δ* 185.5, 181.9, 166.7 (dd, ^*1*^*J*_*C-F*_ = 261.0 Hz, ^*4*^*J*_*C-P*_ = 3.0 Hz), 158.1, 146.0, 138.7, 136.7 (dd, *J* = 11.7, 9.5 Hz), 133.8, 133.1, 132.4, 131.4, 128.4, 128.2, 126.3, 126.0, 118.4 (dd, *J* = 22.7, 14 Hz), 114.0 (dd, ^*1*^*J*_*C-P*_ = 90.2 Hz, ^*4*^*J*_*C-F*_ = 3.7 Hz), 74.0, 43.3, 35.3, 34.5, 30.5, 30.3, 30.0, 29.5, 29.3, 29.3, 25.9, 23.3 (d, *J* = 49.9 Hz), 22.8 (d, *J* = 5.1 Hz).

### 2.5 MitoCF_3_-ATO

In a pear-shaped flask under an inert atmosphere, **ATO-Br** (0.3 g, 0.52 mmol) and tris-(4-trifluoromethylphenyl)-phosphine (0.96 g, 2.05 mmol) were stirred at 90 °C for 288 h. Crude was recovered in CH_2_Cl_2_, dropwise added to Et_2_O (250 mL), and left to precipitate overnight at −20 °C. Et_2_O was removed, and the crude was concentrated under reduced pressure. Purification by flash chromatography on silica gel (Et_2_O 100% to CH_2_Cl_2_ 100%, then CH_2_Cl_2_/EtOH from 0% to 10% EtOH) yielded the corresponding **MitoCF_3_-ATO** as a golden solid (0.08 g, 15% yield).

HR-MS calculated for **MitoCF_3_-ATO** C_53_H_50_ClF_9_O_3_P^+^ [M]^+^ 971.3037, found 971.3028.

^1^H NMR (400.13 MHz, CDCl_3_), *δ* 8.18 (dd, *J* = 12.4, 8.2 Hz, 6H), 8.06 (dd, *J* = 7.5, 1.4 Hz, 1H), 8.02-7.98 (m, 7H), 7.69 (quind, *J =* 7.5 Hz, 1.5 Hz, 2H), 7.25-7.23 (m, 2H), 7.18-7.17 (m, 2H), 4.37-4.33 (m, 2H), 4.30 (t, *J* = 6.6 Hz, 2H), 3.20 (tt, *J* = 12.2, 3.3 Hz, 1H), 2.61 (tt, *J* = 12.1, 3.2 Hz, 1H), 2.17 (qd, *J* = 12.7, 3.3 Hz, 2H), 1.97 (dd, *J =* 13.3, 2.6 Hz, 2H), 1.81 (quint, *J =* 7.1 Hz, 2H), 1.73 (dd, *J* = 13.4, 2.7 Hz, 2H), 1.68-1.67 (m, 2H), 1.61-1.52 (m, 4H), 1.50-1.43 (m, 2H), 1.35-1.27 (m, 8H).

^19^F NMR (376.95 MHz, CDCl_3_), *δ* −63.7.

^31^P NMR (161.98 MHz, CDCl_3_), *δ* 25.9.

^13^C NMR (100.58 MHz, CDCl_3_), *δ* 185.5, 181.9, 158.0, 146.0, 138.7, 137.3, 137.1, 134.8, 134.7 (d, ^2^*J*_*C-P*_ = 10.9 Hz), 133.8, 133.1, 132.3, 131.4, 131.4, 128.3, 128.2, 127.5 (dq, ^3^*J*_*C-P*_ = 12.7 Hz, ^*3*^*J*_*C-F*_ = 3.6 Hz), 126.3, 125.9, 122.6 (q, ^*1*^*J*_*C-F*_ = 274.3 Hz), 121.7 (d, ^*1*^*J*_*C-P*_ = 84.5 Hz), 74.0, 43.3, 35.3, 32.2, 30.5, 30.3, 30.0, 29.5, 29.3, 29.3, 29.2, 25.9, 22.7 (d, *J* = 5.5 Hz), 22.2 (d, *J* = 44.9 Hz).

### 2.6 Cyclic voltammetry

Electrochemical measurements were performed using a BAS 100 potentiostat (Bioanalytical Systems) operated with BAS100W v2.3 software. A three-electrode configuration was used, consisting of a platinum working electrode (1.6 mm diameter), a platinum auxiliary electrode, and a silver (Ag)/silver chloride (AgCl) reference electrode (3 M sodium chloride [NaCl]). All compounds (5 × 10^−4^ M) were analyzed in CH_2_Cl_2_ containing 0.1 M (nBu)_4_NPF_6_ as the supporting electrolyte. The Ag/AgCl electrode was calibrated with ferrocene (E°(Fc/FC^+^) = 0.46 V vs. SCE) [22].

The scan rate was 100 mV/s. Between scans, the platinum working electrode was polished to prevent passivation. Because cyclic voltammetry (CV) does not quantitatively determine the electron stoichiometry of redox events, all current intensities were normalized to the first quinone reduction, assumed to be a one-electron process. Potentials are reported relative to saturated calomel electrode (SCE). When multiple redox processes were observed, repeated scans were collected to evaluate reversibility.

### 2.7 EPR coupled to the spin-trapping technique

Superoxide production in an NADH/xanthine oxidase (XO) system was monitored using the spin trap DIPPMPO. Reaction mixtures contained DIPPMPO (50 mM), DTPA (1 μM), NADH (200 μM), XO (25 mU/mL), and **MitoR-ATO** analogs (100 μM) in 50 mM phosphate buffer (pH 7.4). Formation of the DIPPMPO–OOH adduct was followed by recording the low-field absorption line. Approximately 3 min after initiating the reaction, EPR spectra were acquired at a rate of one full scan per minute for 20 min (20 spectra).

Instrument settings: microwave power 20 mW; modulation amplitude 1 G; smooth point 1; gain 7.5 dB; sweep time 30 s; conversion time 29.3 ms.

### 2.8 Determination of NADH oxidation rate

In the absence of reductant substrates, XO oxidizes NADH using molecular oxygen to generate superoxide. Assays were performed in 96-well plates using air-saturated 50 mM sodium phosphate buffer (pH 7.4) containing DTPA (1 mM), XO (37 mU/mL), and NADH (200 μM). Twelve concentrations of **MitoR-ATO** (two-fold serial dilutions from 100 μM) were tested; DMSO content remained below 5%. Blanks lacking XO or NADH were also included. NADH consumption was monitored at 340 nm (ε = 6.22 × 10^3^ M^−1^ cm^−1^) [23]. Solutions were equilibrated at 37 °C for 5 min, and reactions were initiated by adding XO. Spectra were recorded once per minute for 30 min, with 5-s plate shaking between scans.

Linear reaction rates were obtained from 2 to 15 min and normalized to the blank control. The relative rate of NADH oxidation was plotted against inhibitor concentration.

### 2.9 Cell culture

MiaPaCa-2 (CRL-1420) and MDA-MB-231 (HTB-26) cell lines were obtained from ATCC (Manassas, VA) and routinely authenticated. Cells were maintained at 37 °C in 5% CO_2_ in DMEM (Thermo Fisher, Waltham, USA; Cat# 11965) supplemented with 10% fetal bovine serum. All lines were stored in liquid nitrogen and used within 20 passages after thawing.

### 2.10 Cell proliferation

Cell proliferation was monitored using the IncuCyte Live-Cell Analysis System [4,24,25]. This probe-free, noninvasive imaging platform continuously measures changes in cell confluence as a surrogate marker for proliferation. Cells were seeded at 1000 cells per well (96-well plate, triplicate) and allowed to adhere overnight before treatment with **MitoR-ATO** analogs. Confluence was recorded over several days.

### 2.11 Mitochondrial complex I and III function analysis

For mitochondrial complex-specific activities, the treated cells were acutely permeabilized using the Seahorse XF Plasma Membrane Permeabilizer. Complex I activity was measured using pyruvate (10 mM)/malate (1.5 mM) in the presence of malonate (10 mM). Complex III activity was assessed using duroquinol (0.5 mM) with rotenone (1 μM) and malonate (10 mM). Rotenone, malonate, and antimycin A (Sigma-Aldrich, St. Louis, MO) served as selective inhibitors of complexes I, II, and III. Half-maximal inhibitory concentration (IC_50_) values were determined as described [26,27].

### 2.12 Computational studies, sequence alignment, and docking assessment

Rieske (UniProt: P47985, P13272, Q6CI02, P08067) and cytochrome *b* (UniProt: P00157, P00156, Q9B6D0, P00163) sequences were aligned using Clustal Omega [28]. The mitochondrial complex III X-ray crystal structure, complexed with atovaquone and ubiquinol-6 (PDB 4PD4, 3.0 Å resolution), was prepared using Meeko [29]. Water molecules were removed, hydrogens were added, bond orders were assigned, and the hydrogen-bonding network was optimized. Ligands were assigned Gasteiger charges with Meeko [29]. Docking grids were centered on the **ATO** moiety using Autogrid-4, and docking was performed with AutoDock-GPU [30]. Predicted poses were compared with the crystallographic orientation of **ATO** in 4PD4. Additional cytochrome *bc*_*1*_ structures co-crystallized with **ATO** or stigmatellin A (PDB: 1SQX, 3CX5, 8AB7) were analyzed for cross-species comparison. Figures were generated with PyMOL [31].

The Schrödinger Glide SP (standard precision) protocol was adopted to refine binding-pose predictions and enable seamless integration with Desmond molecular dynamics simulations. Therefore, we switched to Schrödinger's Maestro suite [32].

### 2.13 Protein and ligand preparation

The mitochondrial complex III X-ray crystal structure (PDB 4PD4) was prepared using Schrödinger's Protein Preparation Wizard [33]. Water molecules were removed, hydrogens added, bond orders assigned, and the hydrogen-bonding network was optimized at pH 7.4 using PROPKA, which predicts pKa shifts from the local protein environment, including desolvation and electrostatic effects. Finally, the restrained minimization of the protein was applied using the OPLS4 force field. Ligands were sketched in Maestro, and the LigPrep module was used to generate 3D conformers and perform energy minimization [34].

### 2.14 Grid generation and molecular docking

A grid box large enough to accommodate larger ligands (45 × 45 × 45 Å) was defined around the co-crystallized **ATO** for the Q_o_ site and around the ubiquinol-6 for the Q_i_ site using Glide's Receptor-Grid-Generation tool [35]. All docking settings were set to their default values.

### 2.15 Molecular dynamics

Molecular dynamics (MD) simulations were performed using Desmond in the Schrödinger package [36]. First, the best protein-ligand complexes were retrieved from the docking results. Then, the solvated system for simulation was embedded in a pre-equilibrated POPC bilayer using the System-Builder panel. The solvent model selected was SPC, with an orthorhombic box of dimensions 10 Å. Na^+^ (sodium) and Cl^−^ (chlorine) ions were added to achieve a physiological salt concentration of 0.15 M NaCl, with the overall charge of the system neutralized accordingly. The system was minimized in the minimization panel using the default parameters. Finally, the simulation parameters were set up in the molecular dynamics tool, in which the minimized system was evaluated over a 100 ns simulation time, and the ensemble class was set to isothermal-isobaric (NPT) to maintain constant temperature and pressure at 300K and 1.01325 bar, respectively. Simulations were run with the OPLS4 force field. After running the MD simulation, the generated results were analyzed using the Simulation Interactions Diagram tool. Only interactions that occur more than 30% of the simulation time were considered.

### 2.16 Calculated octanol/water partition coefficients

LogP values for **MitoR-ATO** analogs were obtained using quantitative structure-activity relationship (QSAR)-based physicochemical modeling (see Table S1). A consensus model implemented in ChemAxon software (San Diego, CA) was used to estimate molecular hydrophobicity [37,38].

## 3 Results and discussion

### 3.1 Redox-cycling properties of MitoR-ATO

#### 3.1.1 Redox potentials

**Fig. 3:**
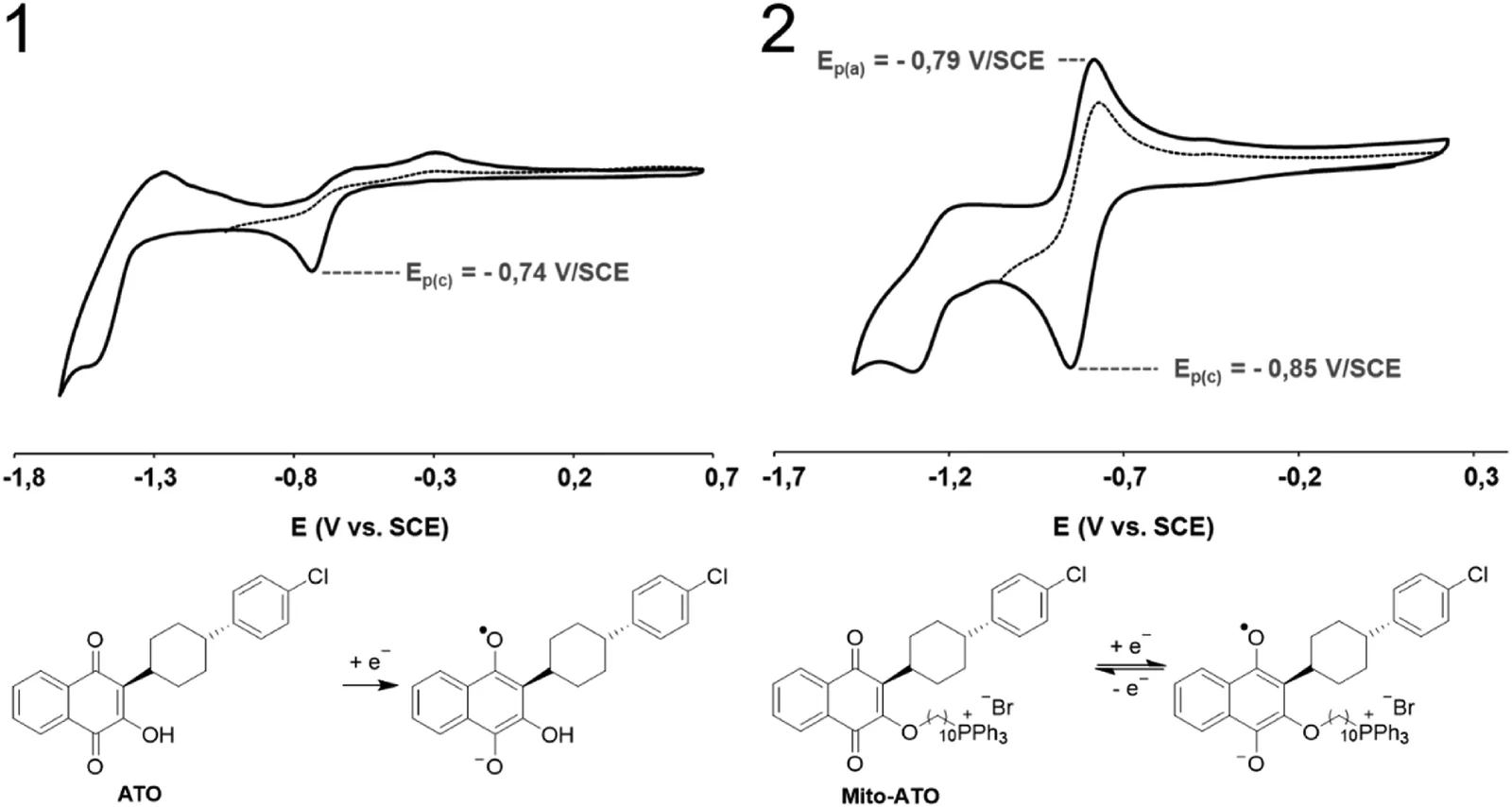
**Cyclic voltammetry experiments of ATO and Mito-ATO.***Panel 1*: Cyclic voltammogram of ATO. *Panel 2*: Cyclic voltammogram of Mito-ATO. The dotted lines represent the reversibility studies for the selected electrochemical processes.

Redox potentials of the **MitoR-ATO** analogs were determined by cyclic voltammetry (Table 1). All values are reported relative to the SCE. **ATO** shows two irreversible one-electron reductions at E_1/2_(red_1_) = −0.71 V/SCE and E_1/2_(red_2_) = −1.47 V/SCE (Fig. 3, Panel 1). In contrast, **Mito-ATO** exhibits a pseudo-reversible one-electron reduction at E_1/2_(red_1_) = −0.82 V/S CE (ΔEp = 78 mV) and an additional irreversible one-electron reduction at E_1/2_(red_2_) = −1.27 V/SCE (Fig. 3, Panel 2). All other mitochondria-targeted agents display voltammetric patterns and redox potentials comparable to those of **Mito-ATO** (Fig. S17–S19, Table S2–S4).

**Table 1:** Redox potential of ATO derivatives against SCE.

	E_1/2_ red_1_		E_1/2_ red_1_
**ATO**	−0,71	**MitoF-ATO**	−0,82
**Mito-ATO**	−0,82	**MitoCH_3_-ATO**	−0,83
**MitoCF_3_-ATO**	−0,82	**O_2_/O_2_^●-^**	−0.38

Results indicate that **Mito-ATO** is slightly more difficult to reduce than **ATO** by ∼110 mV. The first reduction is irreversible for **ATO** because of a probable proton-coupled electron transfer but is reversible for **Mito-ATO**, and this reversibility is maintained across other substituted analogs (Fig. S17–S19, Table S2–S4) [39]. These results reveal that the nature of the substitution does not affect the redox potential of **MitoR-ATO** analogs. However, they do show that alkylation of the free hydroxyl moiety of **ATO** is essential for the reversibility of the first reduction process.

#### 3.1.2 EPR coupled to the spin-trapping technique

**Fig. 4:**
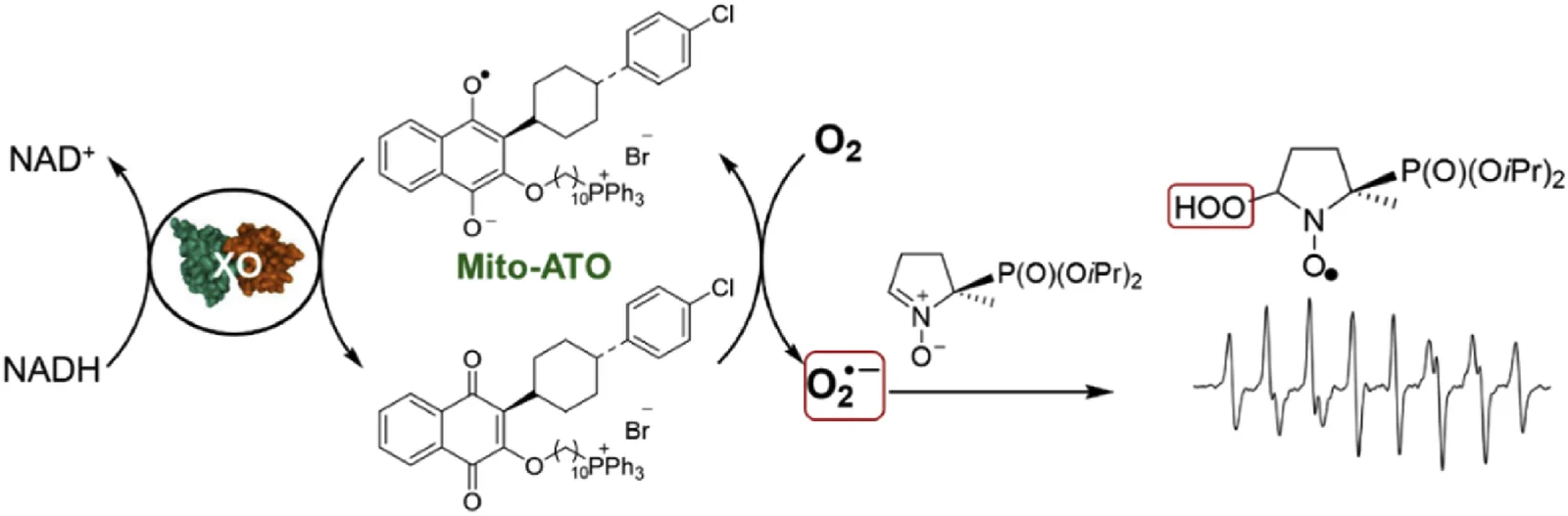
Detection of superoxide formed from the redox-cycling of Mito-ATO.

EPR spin-trapping experiments were conducted in an NADH/XO system using the phosphorylated nitrone DIPPMPO as the spin trap (Fig. 4). DIPPMPO reacts with superoxide to form a persistent DIPPMPO-OOH adduct with a characteristic EPR signature.

**Fig. 5:**
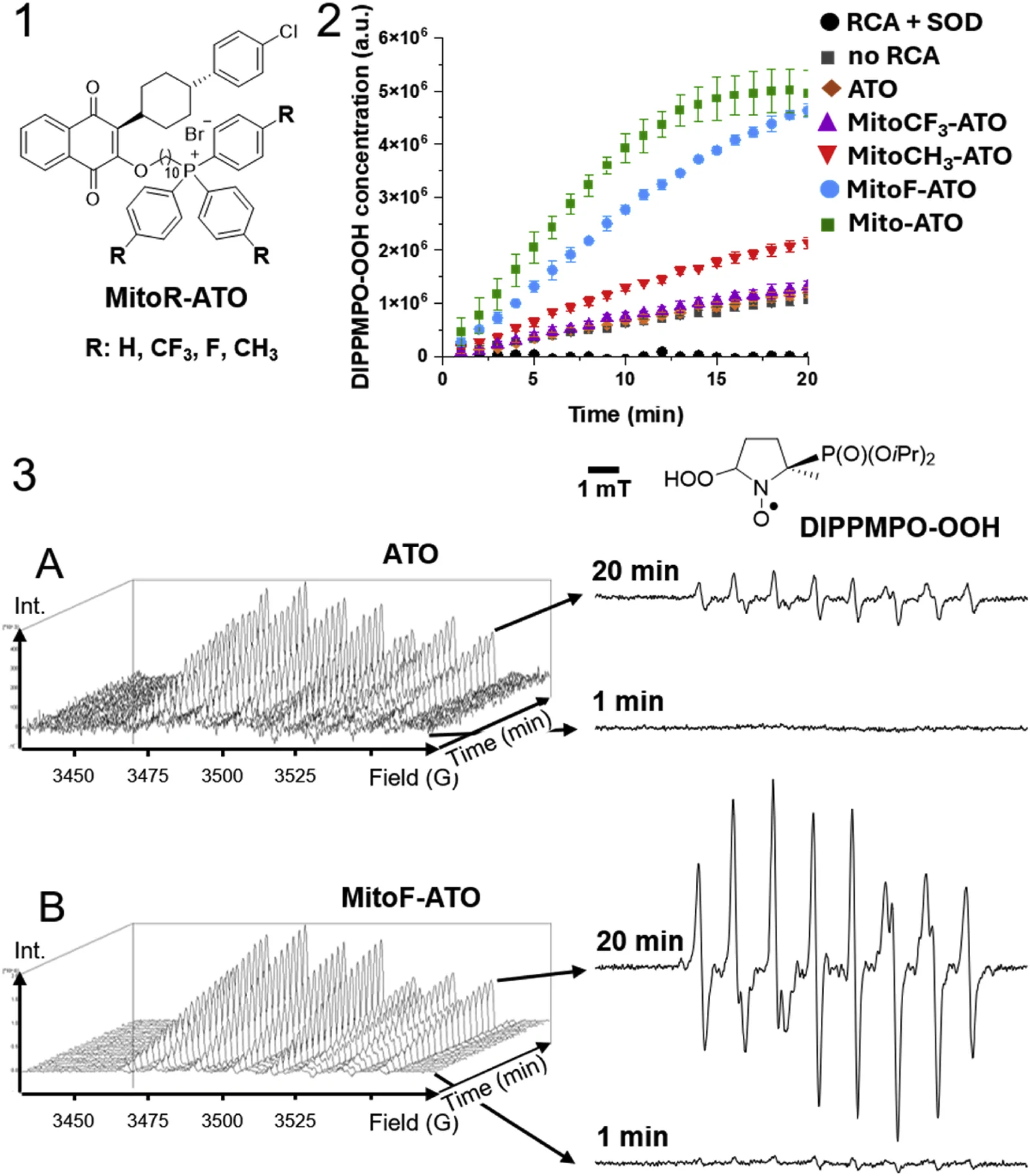
**Redox-cycling activity of MitoR-ATO analogs.***Panel 1:* Chemical structures of Mito-ATO, MitoCF_3_-ATO, MitoF-ATO, and MitoCH_3_-ATO. *Panel 2:* Growth curves of the DIPPMPO-OOH adduct under the following conditions: (●) with redox-cycling agent (RCA) + SOD (600 U/mL); (■) without RCA (); ATO (100 μM) (); MitoCF_3_-ATO (100 μM) (); MitoCH_3_-ATO (100 μM) (); MitoF-ATO (100 μM); and () Mito-ATO (100 μM). *Panel 3:* 2D EPR spectra recorded after 1 min incubation in a reaction mixture containing NADH (0.2 mM), XO (25 mU/mL), DTPA (1 mM), and DIPPMPO (50 mM) in phosphate buffer (0.05 M, pH 7.4). Spectra were recorded at a rate of 1 full scan per minute for 20 min, resulting in 20 consecutive spectra. *Panel 3A:* spectra obtained with ATO (100 μM, –). Associated EPR spectra extracted from the 2D scan, corresponding to 1 min and 20 min of incubation, are shown. *Panel 3B:* corresponding spectra in the presence of MitoF-ATO (100 μM, –). Associated EPR spectra extracted from the 2D scan, corresponding to 1 min and 20 min of incubation, are shown. Additional spectra are provided in the Supplementary Information. Data are mean ± SD, n = 3.

The DIPPMPO-OOH signal intensities (Table S5) reflect the redox-cycling activity of the **Mito-ATO** derivatives over time (Fig. 5 Panel 2, Fig. S23–S27). **ATO** produced superoxide levels similar to the control (Fig. 5 Panel 2, Fig. S22). **Mito_16_-ATO** behaved similarly (Fig. S24), consistent with previous findings [20].

The –CF_3_ substituent markedly suppressed redox-cycling activity (Fig. S27), while the –CH_3_ substituent slightly increased superoxide production relative to **ATO** (Fig. S25). The –F substituent produced a pronounced increase in DIPPMPO-OOH formation, yielding redox-cycling activity equal to or greater than that of the unsubstituted **Mito-ATO** (Fig. S23). Follow-up experiments at lower concentrations (25–50 μM) confirmed that **MitoCF_3_-ATO** does not exhibit detectable redox cycling under these conditions (Fig. S29–S30). Importantly, the addition of SOD (600 U/mL) before activation abolished all EPR signals, confirming that the observed spectra arise specifically from superoxide trapping (Fig. S21). EPR-spin trapping results showed that TPP^+^ substituents alter the redox cycling effects of the parent molecule, **ATO**, with respect to superoxide generation.

#### 3.1.3 NADH oxidation kinetics

**Fig. 6:**
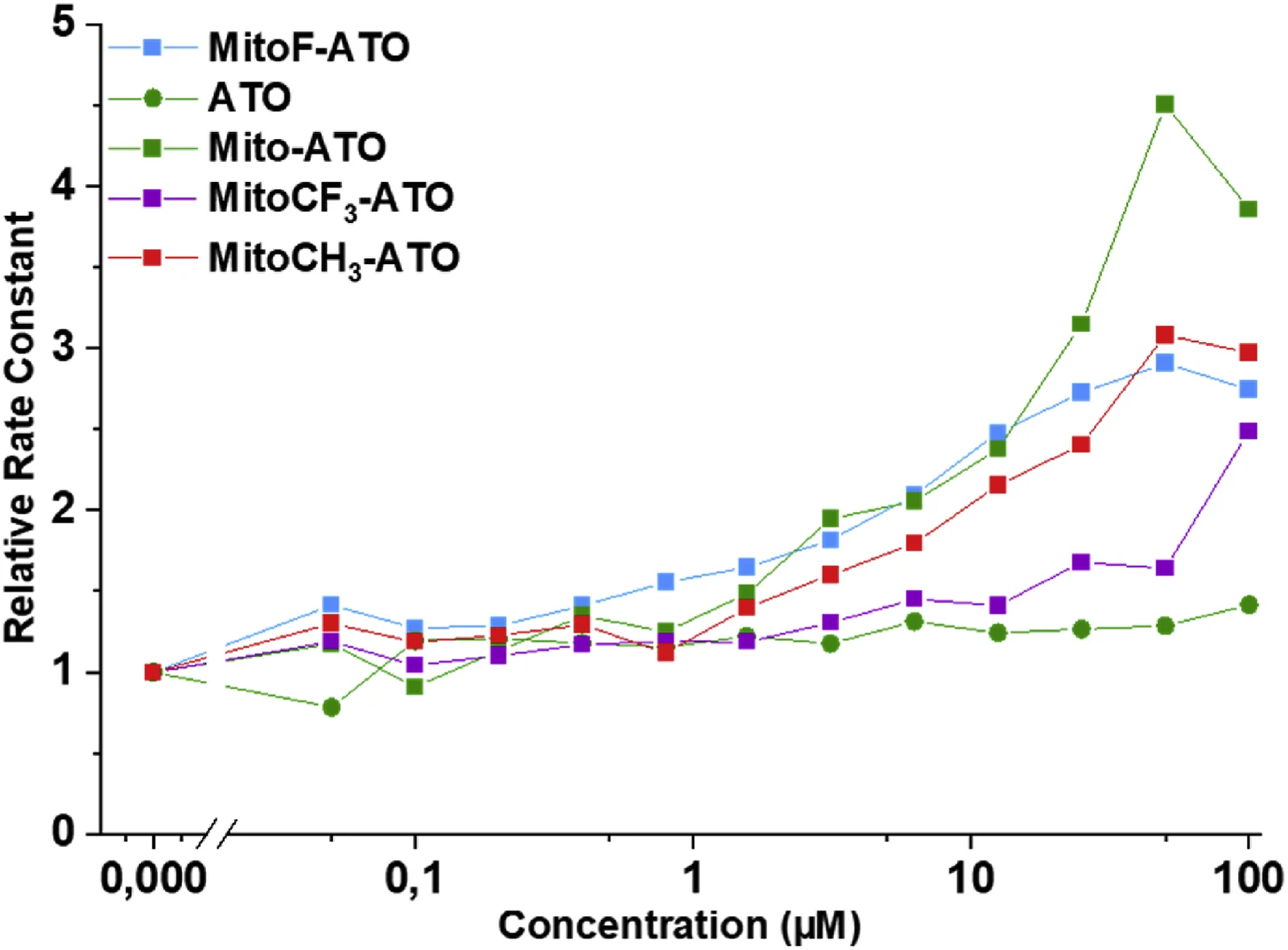
**Concentration-dependent effect of MitoR-ATO on the rate of NADH oxidation.** Reactions included XO (0.037 U/mL), NADH (200 μM), DTPA (1 mM), and 50 mM phosphate buffer (pH 7.4) in the presence of 0–100 μM MitoR-ATO (Fig. S16–S20). Observed rate has been normalized to the initial rate under these conditions in the absence of MitoR-ATO (dotted line). Data shown are the mean ± SD, n = 3.

The redox-cycling ability of quinone-type electron acceptors (**ATO**, **Mito-ATO**, and analogs) was assessed by monitoring the oxidation of NADH in the XO/NADH system. As shown in Fig. 6, the **MitoR-ATO** analogs exhibit trends consistent with the EPR results obtained with the spin-trapping technique. **ATO** and **MitoCF_3_-ATO** induce minimal increases in NADH oxidation, whereas **Mito-ATO** produces the highest rate of oxidation.

### 3.2 Cell proliferation

**Fig. 7:**
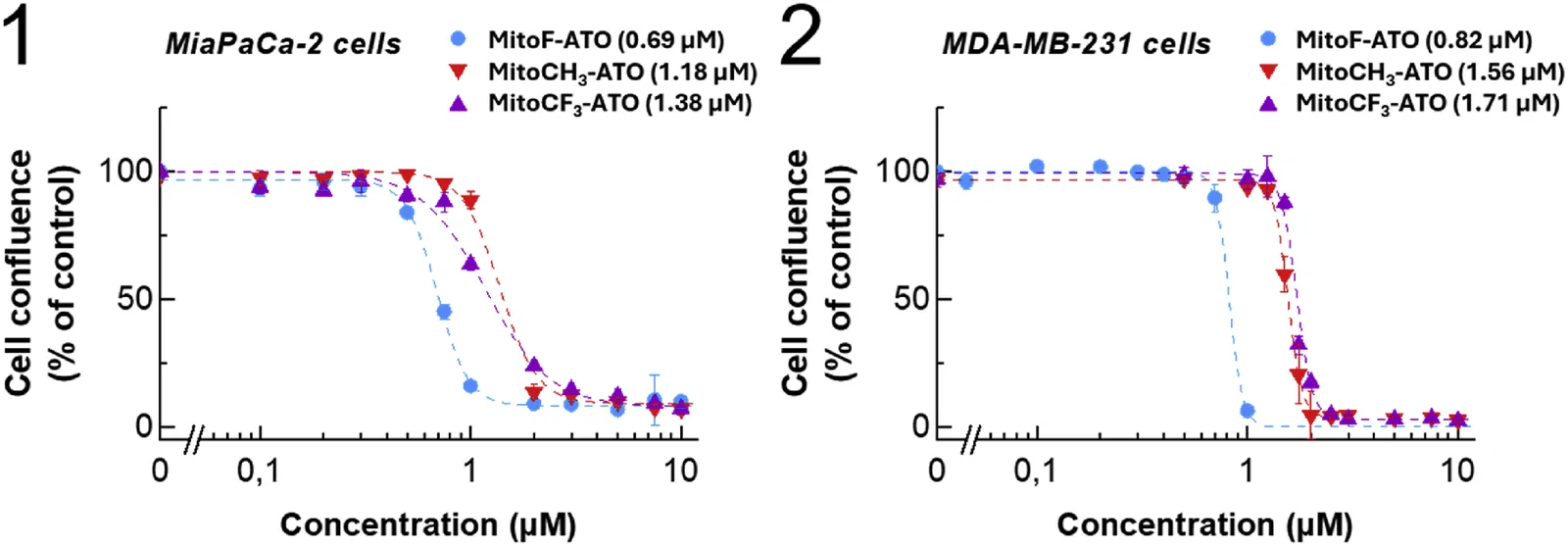
**Effects of MitoF-ATO, MitoCH_3_-ATO, and MitoCF_3_-ATO on the proliferation of two cancer cell lines.***Panel 1* shows relative confluence (control i = 100%) plotted as a function of compound concentration for the proliferation of MiaPaCa-2 pancreatic cancer cell line. Dashed lines represent nonlinear regression curves used to drive the IC_50_ values. Data are presented as mean ± SD, n = 4. *Panel 2* is the same as Panel 1, but for the proliferation of the MDA-MB-231 breast cancer cell line.

To investigate how substituents in TPP^+^ influence their biological effects, we evaluated the antiproliferative properties of the **MitoR-ATO** analogs in MiaPaCa-2 (pancreatic cancer) and MDA-MB-231 (breast cancer) cells using the IncuCyte Live-Cell Analysis System. For MiaPaCa-2 cells, the IC_50_ values of **MitoF-ATO**, **MitoCH_3_-ATO**, and **MitoCF_3_-ATO** were 0.69 μM, 1.18 μM, and 1.38 μM, respectively. In comparison, the IC_50_ of **Mito-ATO** is 0.22 μM for this cell line [20]. Both compounds inhibited cell proliferation in a dose-dependent manner (Fig. 7, Panel 1, Fig. S36). In MDA-MB-231 cells, the IC_50_ values were 0.82 μM for **MitoF-ATO**, 1.56 μM for **MitoCH_3_-ATO**, and 1.71 μM for **MitoCF_3_-ATO** (Fig. 7, Panel 2, Fig. S37). In comparison, the IC_50_ of **Mito-ATO** is 0.32 μM for this cell line [20].

### 3.3 Mitochondrial complex I and III activity measurements

**Fig. 8:**
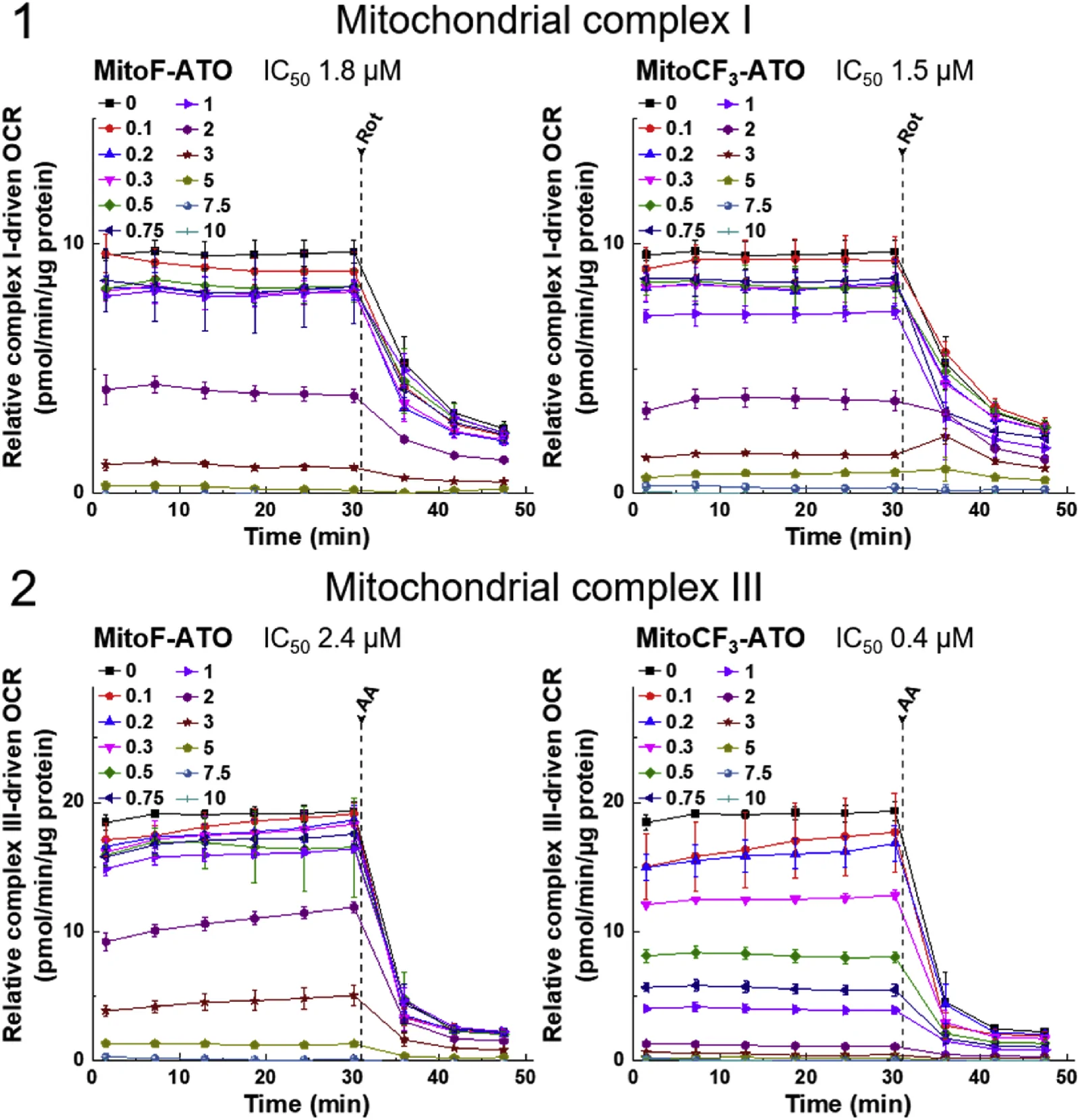
**Effects of MitoR-ATO analogs on the oxygen consumption induced by mitochondrial complexes I and III.** Dose-dependent effect of MitoF-ATO (left) or MitoCF_3_-ATO (right) on Complex I dependent OCR (panel 1) and complex III-dependent OCR (panel 2) was measured in MiaPaCa-2 cells. Relative complex I (1.5 mM malate, 10 mM pyruvate, 10 mM ADP)-driven OCR (panel 1) and relative complex III (0.5 mM duroquinol, 10 mM ADP)-driven OCR (panel 2) were monitored by XF-96 analyzer. Either Rot (complex I inhibitor) or antimycin A (AA, complex III inhibitor) was acutely added and OCR assayed immediately. The mitochondrial complex I (panel 1)- and III (panel 2)-driven OCR (calculated as Rot or AA inhibitable OCR) are plotted against the concentration of MitoR-ATO analogs for determination of the IC_50_ values.

We next evaluated the effects of **MitoR-ATO** analogs on mitochondrial complex activities using complex I- and complex III-driven oxygen consumption assays (Fig. 8). Both **MitoCF_3_-ATO** and **MitoF-ATO** inhibited complex I- and complex III-dependent oxygen consumption. Notably, **MitoCF_3_-ATO** displayed greater specificity toward complex III inhibition, with IC_50_ values of 0.4 μM (complex III), in comparison to **MitoF-ATO** with an IC_50_ of 1.5 μM. Previously reported IC_50_ values for **Mito-ATO** are 0.32 μM and 0.78 μM for complex I- and complex III-driven OCR, respectively [20].

Based on OCR experiments, we can conclude that electron-withdrawing and electron-donating substituents on TPP^+^ in **Mito-ATO** do not markedly affect the dose-response characteristics. However, increasing the alkyl side-chain length in **Mito-ATO** significantly affects its ability to inhibit mitochondrial complex III-driven OCR. For example, **Mito_16_-ATO** did not inhibit mitochondrial complex III-driven OCR compared with **Mito-ATO** (**Mito-ATO** IC_50_ = 0.78 μM, **Mito_16_-ATO** IC_50_ > 10 μM) [20].

### 3.4 Computational analysis of MitoR-ATO analogs at the mitochondrial complex III site

#### 3.4.1 Computational analysis of ATO binding at the MCIII Q_o_ site

**Fig. 9:**
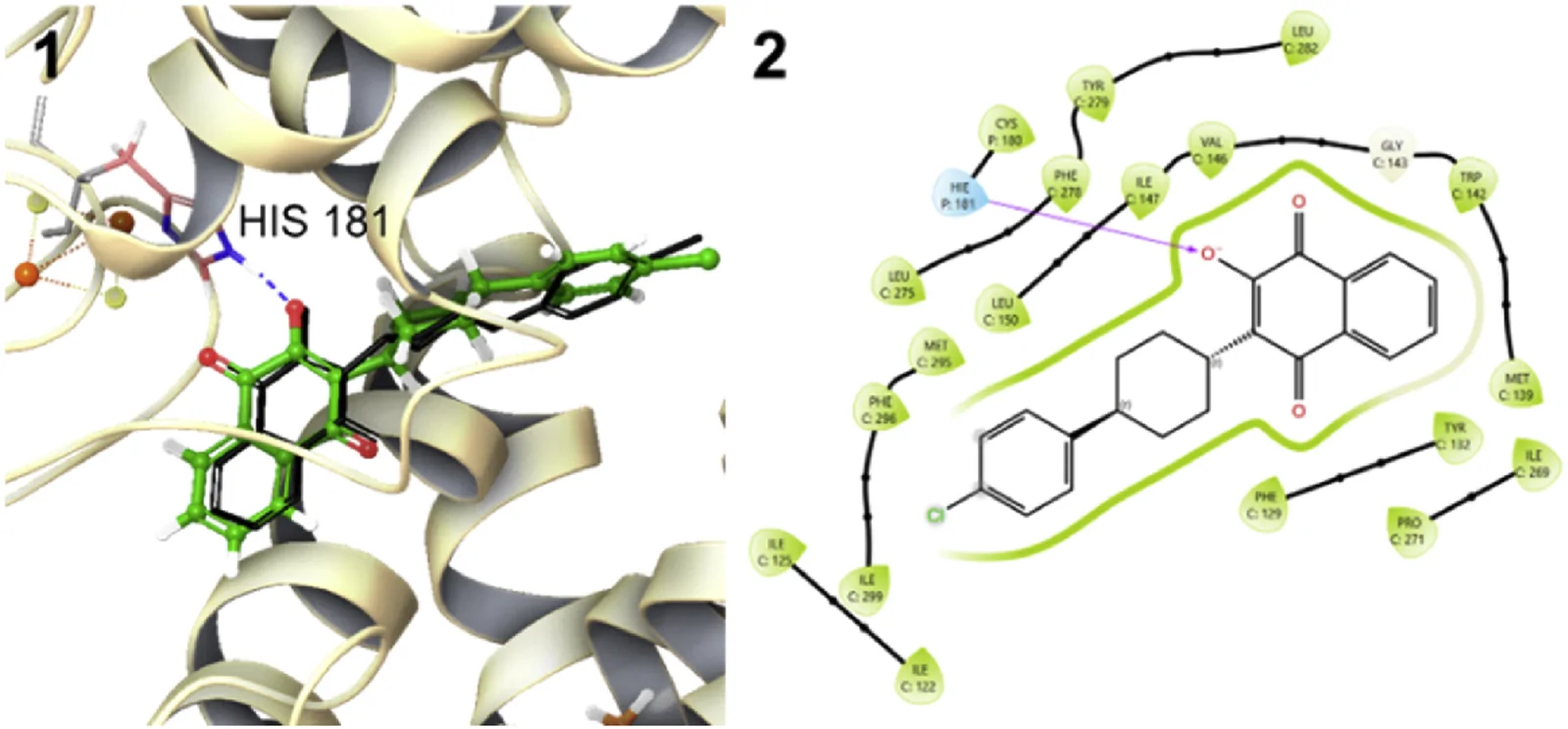
**ATO binding to complex III Q_o_ site.***Panel 1:* Calculated docked ATO pose (green) overlapping crystallized ATO (black) in 4PD4 Q_o_ site (top-ranked pose, score: −11.2 kcal/mol). *Panel 2:* Calculated docked ATO pose interactions diagram (H-bond involved His181 in blue).

To investigate how **ATO** interacts with mitochondrial complex III, we first reproduced its reported crystallographic binding pose at the Q_o_ site (Fig. 9) [40]. Using these structural data as a reference, we generated a docking model consistent with the reported crystallographic orientation.

Autodock-GPU docking simulations recapitulated the same orientation, including the key hydrogen bond between the ionized **ATO** hydroxyl group and His181. This pose was part of a large, well-defined cluster (93 poses; average score −11.0 kcal/mol), supporting the robustness of the model.

To assess the robustness of our docking approach across species, we repeated the docking analysis using cytochrome *bc*_*1*_ structures from *Yarrowia lipolytica* (PDB 8AB7) and bovine heart mitochondria (PDB 1SQX). In both cases, the predicted binding orientations were highly consistent with their respective crystallized ATO-bound structures (Fig. S40), confirming cross-species reliability.

As an additional control, we evaluated stigmatellin A, a well-characterized inhibitor of the cytochrome *bc*_*1*_ complex [41]. Docking studies in *Saccharomyces cerevisiae* (PDB 3CX5), a strain known to be resistant to **ATO**, reproduced the expected binding mode of stigmatellin A, consistent with its crystallized form. Importantly, using the same ATO-resistant *S. cerevisiae* (3CX5) structure, our model correctly predicted *no binding* of **ATO** to the Q_o_ site, demonstrating both the specificity and predictive value of our docking strategy (Fig. S41–S42).

To exclude potential scoring-function bias, we independently validated these results using Schrödinger Glide. Glide docking identified a binding pose for **ATO** within mitochondrial complex III, at the Q_o_ site (PDB 4PD4) that was highly consistent with the Autodock-GPU model, with a comparable docking score (– 11.2 kcal/mol). This concordance indicates that the observed binding mode is independent of the scoring algorithm employed. Based on this cross-validation, all subsequent docking and simulations were performed using the Schrödinger suite.

**Fig. 10:**
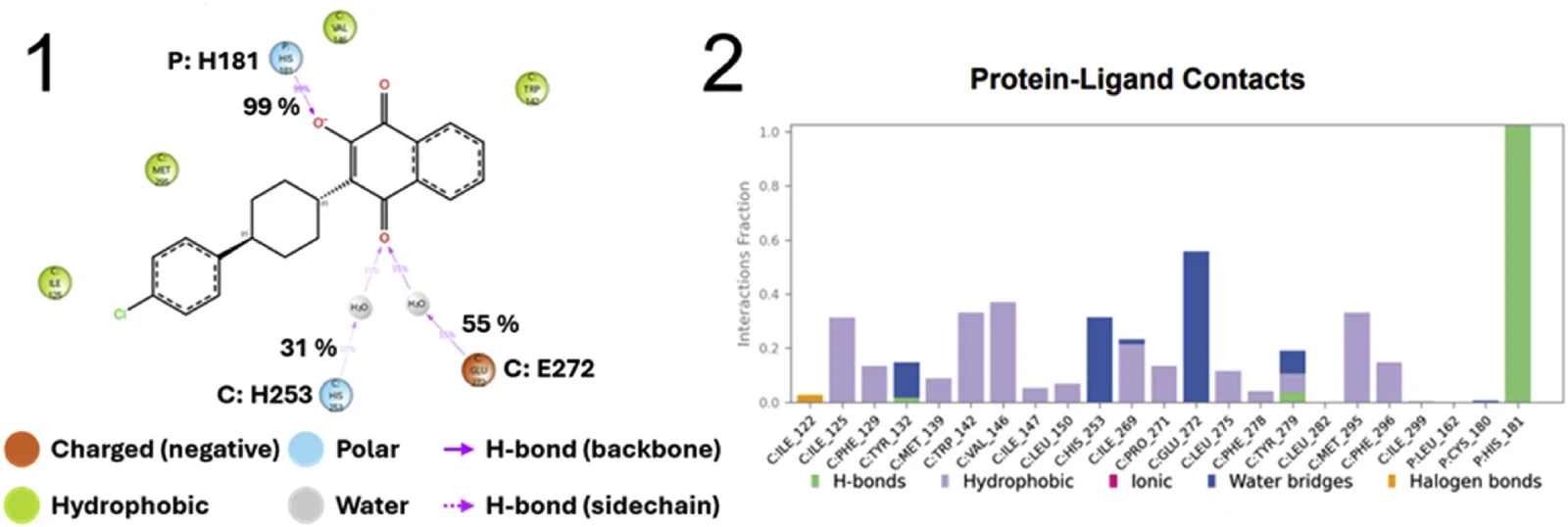
**Interaction profile and protein-ligand contact profile over a 100 ns MD simulation based on the docked pose of ATO.***Panel 1*: Docked ATO pose interactions with protein residues over the 100 ns MD simulation [hydrophobic residues are in green (), polar residues in blue (), negatively charged residues in orange (); backbone H-bonds are represented by solid pink arrows (**−**), side-chain H-bonds by dashed pink arrows (−−)]. *Panel 2*: The protein-ligand contacts per residue index over the 100 ns MD simulation.

Finally, we evaluated the stability of the predicted **ATO** binding mode by performing Desmond molecular dynamics simulations on the top-ranked pose. Because the cytochrome *bc*_*1*_ complex is a transmembrane protein, the system was embedded in a lipid bilayer. Throughout the simulation, **ATO** remained correctly positioned within the Q_o_ site, maintaining the key hydrogen bond with His181 for 99% of the trajectory and with a ligand RMSD of 1.2 Å after alignment on the protein's Cα atoms, thereby supporting the validity and stability of the docked complex (Fig. 10, Fig. S43–S45).

With the model validated, we next probed the binding poses of our **MitoR-ATO**. Despite its known inhibition of mitochondrial complex III (MCIII)-driven oxygen consumption, no satisfying poses were identified for **Mito-ATO** with a reasonable docking score and good overlap with the calculated **ATO**. This lack of docking compatibility likely arises because the TPP ^+^ -targeting moiety is introduced on the **ATO** hydroxyl group that normally forms a deep, stabilizing interaction with His181. This constraint forces TPP ^+^ to fold back over the **ATO** pharmacophore, resulting in an energetically unfavorable configuration. Thus, contrary to our initial hypothesis, **Mito-ATO** and Mito-derivatives likely inhibit MCIII activity through mechanisms independent of Q_o_-site binding. Q_i_-site docking and MD simulations were therefore pursued to explore alternative interactions.

#### 3.4.2 Computational analysis of MitoR-ATO binding at the MCIII Q_i_ site

During the Q-cycle, electrons derived from ubiquinol oxidation at the Q_o_ site are transferred via the low-potential *b-heme* pathway to reduce a bound ubiquinone, such as UQ6 (PDB 4PD4) at the Q_i_ site to ubiquinol [42]. Interactions between ubiquinone and Asp229 or His202 are critical. Those ensure that ubiquinone is properly positioned at the reduction site to accept two electrons and two protons after the Q-cycle's double turnover [43].

Antimycin A was used as a reference to evaluate the accuracy of our docking model at the Q_i_ site. Therefore, we redocked it using the crystal structure from PDB 1PPJ as a reference. The docking procedure on 4PD4 successfully reproduced the experimental pose of Antimycin A observed in PDB 1PPJ (docking score: −8.6 kcal/mol). The redocked pose established the expected interactions within the site by forming two direct H-bonds with Asp229, one through the amine moiety and the other through the neighboring hydroxyl group. These interactions confirm the transferability of our docking model to the Q_i_ site.

**Fig. 11:**
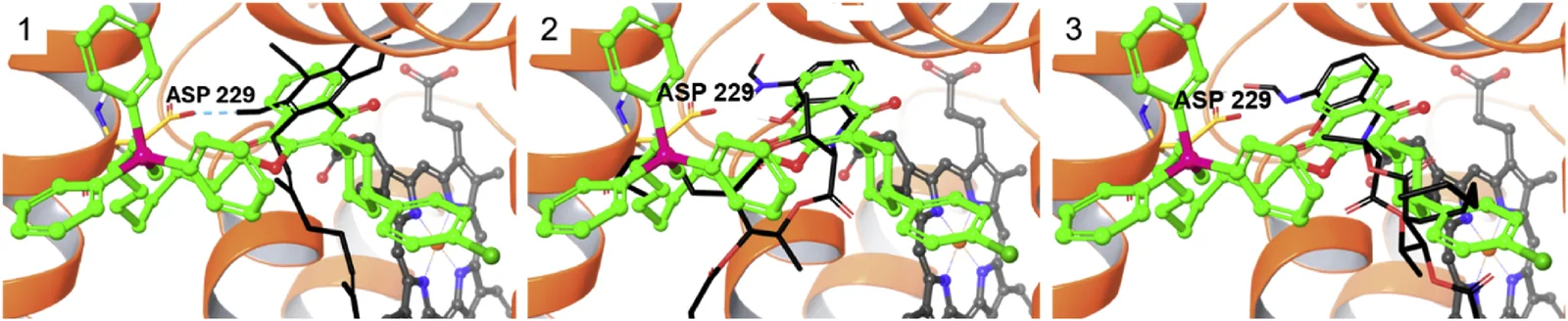
*Panel 1*: Docked pose of Mito-ATO (green) overlapping crystallized UQ6 (black) in 4PD4 Qi site (top-ranked pose score: −7.4 kcal/mol). *Panel 2*: Docked pose of Mito-ATO (green) overlapping docked pose of Antimycin A (black). *Panel 3*: Docked pose of Mito-ATO (green) overlapping crystallized Antimycin A (black), coordinates obtained from PDB 8AB7.

Molecular docking of **Mito-ATO** (docking score: −7.4 kcal/mol) into the Q_i_ site predicted stable poses within the same region occupied by Antimycin A (docked and crystallized, Fig. 11, Panels 2 and 3, Fig. S46) and the natural substrate ubiquinone (Fig. 11, Panel 1). The naphthoquinone moiety is close enough to the porphyrin ring of heme *bH* to allow electron flow (5.9 Å) [42,44]. Despite not maintaining any H-bond with Asp229, **Mito-ATO**'s naphthoquinone remains in proximity (2.87 Å) to allow electrostatic interactions. This docking model was also applied to **Mito_16_-ATO** (docking score: −9.2 kcal/mol), **MitoF-ATO** (−8.8 kcal/mol), **MitoCF_3_-ATO** (−8.7 kcal/mol), and **MitoCH_3_-ATO** (−8.6 kcal/mol). Those four molecules share the same general remarks regarding the chemical environment as **Mito-ATO**. **Mito_16_-ATO** has a 16-carbon linker and has previously been described as inactive toward mitochondrial complex III, and will serve here as a negative control [20]. **MitoR-ATO** analogs show a slightly better docking score than **Mito-ATO**, most likely due to the nature of the substitution on the TPP^+^ moiety and, for **Mito_16_-ATO**, an increased number of hydrophobic contacts.

All five docked systems were subjected to 100-ns molecular dynamics simulations. Cytochrome *bc*_*1*_ Cα atoms RMSD values converged between 3.5 Å and 4.8 Å across all systems; this is expected for a large membrane-embedded complex (Figs. S47, S50, S53, S56, and S59). Ligand RMSD values converged, ranging from 4.8 Å (**MitoCF_3_-ATO**) to 7.2 Å (**Mito_16_-ATO**), indicating stable binding patterns throughout the simulation. These higher RMSD values might be due to the tested compounds all having a long, flexible linker that tends to collapse themselves during docking and then extend during molecular dynamics simulations. The naphthoquinone moiety retained a stable geometry according to the RMSF values per atom index regardless of the linker length and substitution of the TPP^+^.

Therefore, it remained in close enough contact to the porphyrin ring of heme *bH* to allow electron flow (see Supplementary Information, Fig. S46–S60).

**Fig. 12:**
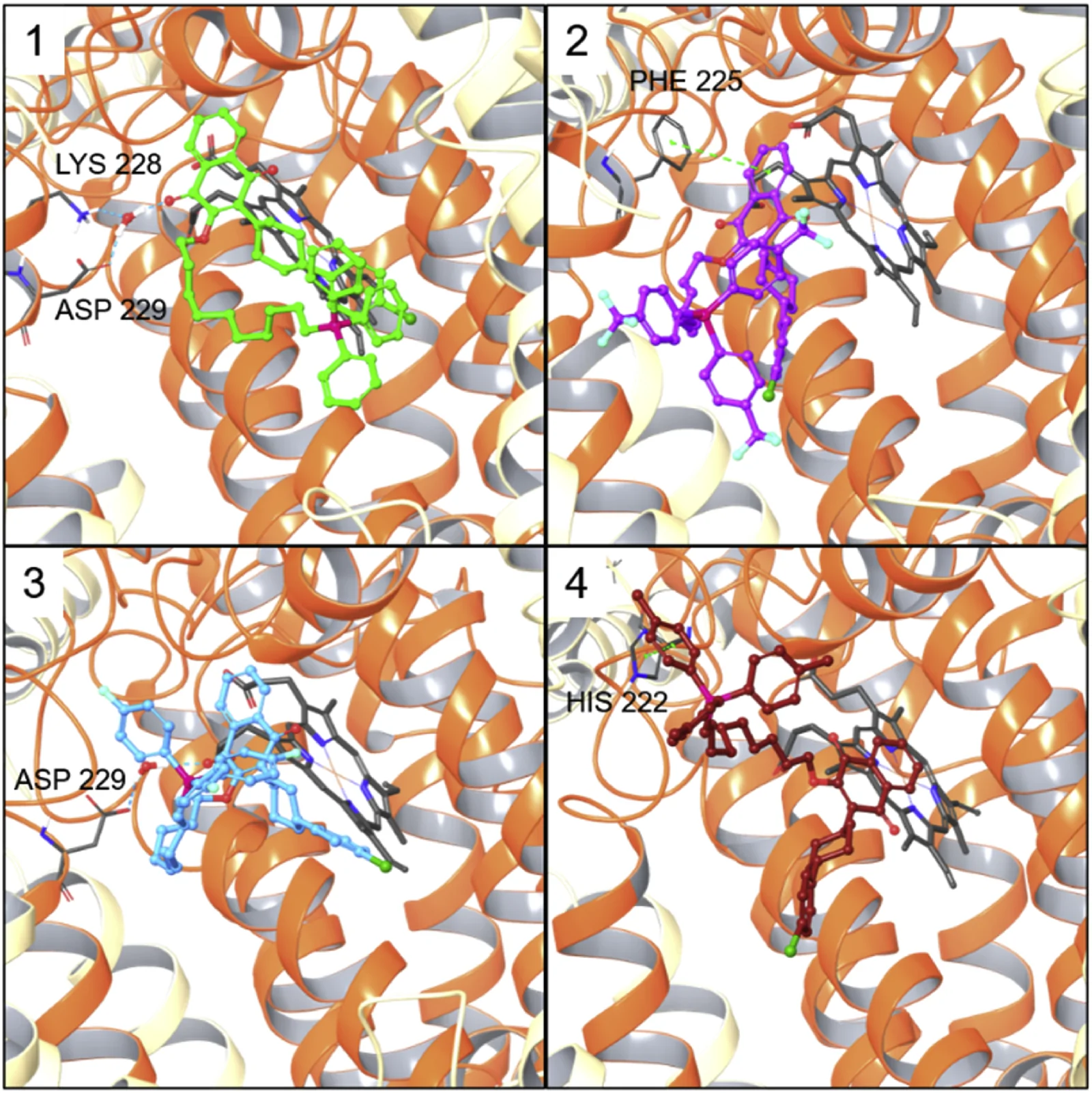
**Selected poses after the 100 ns MD simulations for Mito-ATO (Panel 1), MitoCF_3_-ATO (Panel 2), MitoF-ATO (Panel 3), and MitoCH_3_-ATO** (Panel 4). Water-bridged interactions are displayed in blue, aromatic interactions in green. Key residues and heme *bH* are in grey.

**Fig. 13:**
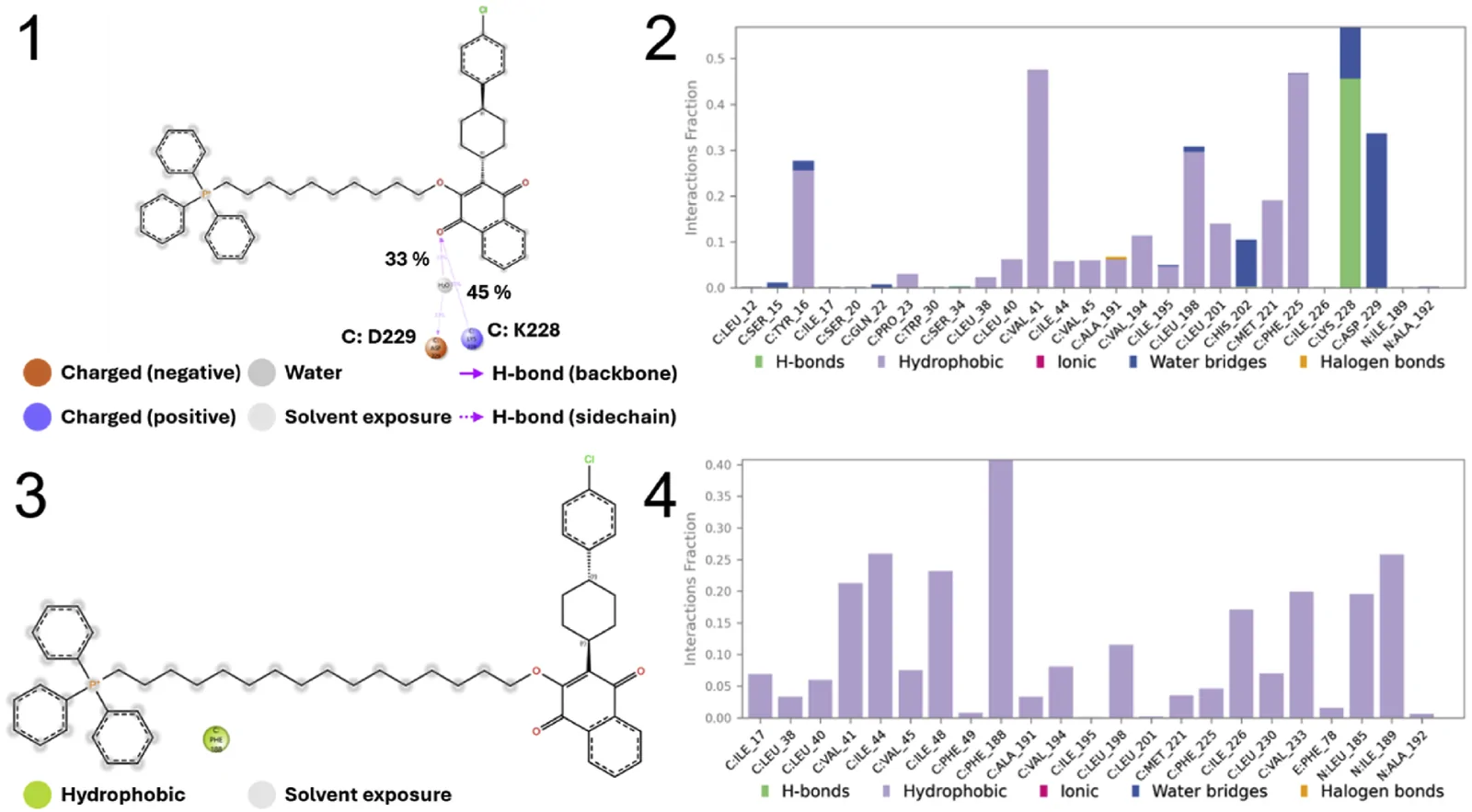
**Interaction profile and protein-ligand contact profiles over a 100 ns MD simulation based on docked Mito-ATO and Mito_16_-ATO.***Panel 1*: Docked Mito-ATO pose interactions with protein residues at least 30% of the time of the 100 ns MD simulation (positively charged residues are represented in blue (), negatively charged residues in orange (); backbone H-bonds are represented by solid pink arrows (**−**), side-chain H-bonds by dashed pink arrows (**−−**)). *Panel 2*: Protein-ligand contacts per residue index over the 100 ns MD simulation. *Panel 3*: Docked Mito_16_-ATO pose interactions with protein residues at least 30% of the time of the 100 ns MD simulation (hydrophobic residues are represented in green). *Panel 4*, Protein-ligand contacts per residue index over the 100 ns MD simulation [hydrophobic residues are in green ()].

Among the five compounds, **Mito-ATO** (MCIII-driven OCR IC_50_ = 0.8 μM) [20] most closely resembles the native ubiquinone and Antimycin binding mode at the Q_i_ site (Fig. 12, panel 1). Over the course of the simulation, it formed H-bonds with Lys228 (45% of the simulation time) and water-bridged contacts with Asp229 on chain C (30%), as well as minor water-bridged contacts with His202 on chain C (10%). This suggests that **Mito-ATO** might be amenable to productive reduction via a network of electrostatic interactions (Fig. 13, Panels 1 and 2). In contrast, **MitoCF_3_-ATO** (MCIII-driven OCR IC_50_ = 0.4 μM) could achieve similar potency by relying on dual aromatic contacts with Trp457 on chain A (π-π stacking 31% and π-cation 25% of the simulation time) because of the electron-withdrawing –CF_3_ groups on the TPP ^+^ moiety. It also relies on a punctual π-π stacking between the naphthoquinone moiety and Phe225's side chain on chain C (roughly 20% of the simulation time) (Fig. 12, Panel 2, Fig. S51). **MitoF-ATO** (MCIII-driven OCR IC_50_ = 2.4 μM) displayed only sparse water bridges with residues on chain C (less than 10% of the simulation time) and no dominant contacts (Fig. 12, panel 3, Fig. S57). **MitoCH_3_-ATO** can also stabilize within the Qi site, albeit slightly closer to the heme b*H* moiety, with its naphthoquinone shifted by 90° relative to the other compounds. It remains stabilized through polar contacts between the TPP^+^ targeting moiety and His222's side chain on chain C (30% of the simulation time), between the naphthoquinone and His197's side chain on chain C (74%), and sparse water-bridged interactions with Tyr16 and Ser20 on chain C (Fig. 12, Panel 4, Fig. S55). Finally, **Mito_16_-ATO** showed no productive interactions throughout the simulation, consistent with its status as a negative control for mitochondrial complex III inhibition (Fig. 13, panels 3 and 4; Fig. S60) [20].

**Fig. 14:**
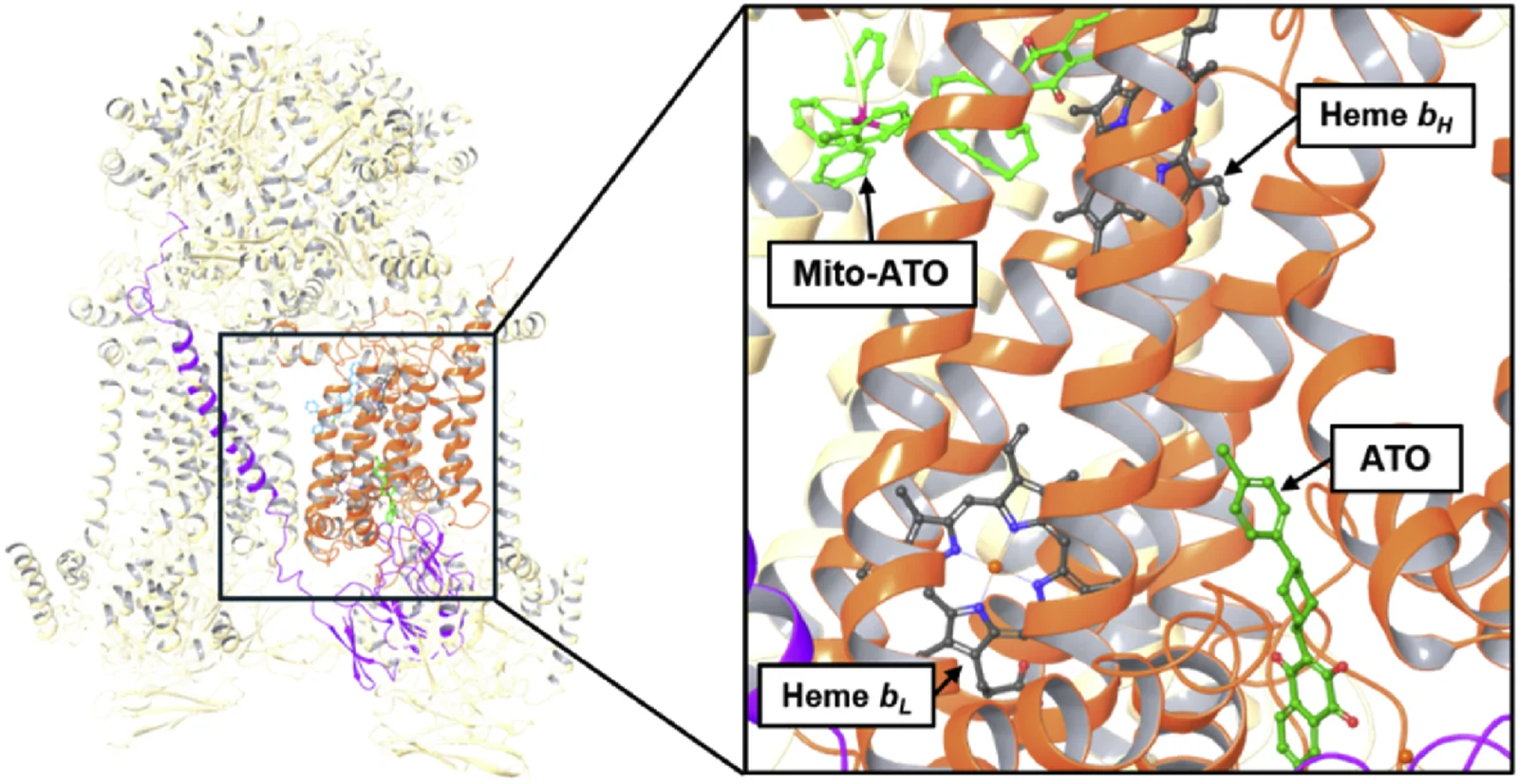
**Q-sites of mitochondrial complex III (PDB:**4PD4**) after the 100 ns MD simulation.** Close-up view showing the Q_i_ site occupied by Mito-ATO and the Q_o_ site occupied by ATO. Hemes *b*_*L*_ (low potential) and *b*_*H*_ (high potential) are shown as cofactors mediating electron transfer across the membrane.

Collectively, the computational results using **ATO** and **Mito-ATO** analogs revealed the following. Firstly, we confirm that **ATO** binds to mitochondrial complex III at the Q_o_ site, therefore validating our computational model. In contrast, **Mito-ATO** binds to the Q_i_ site of mitochondrial complex III, not at the Q_o_ site (Fig. 14). Finally, the longer-chain analog **Mito_16_-ATO** did not bind productively to either the Q_o_ or the Q_i_ site. This is consistent with the reported lack of inhibition of mitochondrial complex III-driven oxygen consumption by **Mito_16_-ATO** [20].

#### 3.4.3 Structure-activity analysis

The relationships among electronic substituents, redox properties, ROS generation, complex III inhibition, and antiproliferative activity are summarized in Table S6. Despite a systematic comparison of these parameters, no simple or direct structure–activity relationship emerged.

Electrochemical analyses showed that electronic substitution of the TPP^+^ moiety had little effect on the intrinsic redox potential or reversibility of the quinone scaffold. In contrast, EPR spin trapping and NADH oxidation assays demonstrated marked differences in redox-cycling efficiency and superoxide generation among the derivatives. However, these variations did not correlate with mitochondrial complex III inhibition or antiproliferative activity, indicating that altered redox cycling alone is insufficient to account for the observed biological effects. Thus, the biological activity of the MitoR-ATO derivatives cannot be predicted solely from the electronic properties of the TPP^+^ substituents.

Molecular docking and molecular dynamics simulations provide a structural basis for this apparent disconnect by revealing distinct ligand–protein interactions within the complex III Qi site. Collectively, these findings demonstrate that, although electronic effects strongly influence the redox behavior of **MitoR-ATO** derivatives, structural determinants governing target engagement are the primary drivers of complex III inhibition and antiproliferative activity. These results underscore the importance of integrating both electronic and structural analyses to understand the mechanism of action and to guide the rational design of next-generation mitochondria-targeted agents.

### 3.5 Therapeutic implications

**Mito-ATO** derivatives have significant potential to enhance tumor responses to radiotherapy by simultaneously alleviating tumor hypoxia and increasing oxidative stress. Tumor hypoxia is a major determinant of radioresistance, and decreasing mitochondrial oxygen consumption has emerged as an effective strategy to improve intratumoral oxygenation and radiosensitivity [45]. In this context, TPP^+^-conjugated compounds selectively accumulate within tumor cell mitochondria, inhibit mitochondrial respiration, and thereby function as effective radiosensitizers [46,47]. Consistent with this mechanism, mitochondria-targeted agents such as Mito-metformin, Mito-lonidamine, and Mito-Q have demonstrated enhanced radiosensitizing activity in multiple preclinical tumor models [12,46,47].

Redox-active quinones provide an additional and complementary mechanism for tumor-selective radiosensitization. β-Lapachone, for example, is metabolized by NAD(P)H:quinone oxidoreductase 1 (NQO1; DT-diaphorase), initiating a futile redox cycle that generates hydrogen peroxide and other ROS [48]. Radiation further induces NQO1 expression, amplifying ROS production and selectively increasing oxidative damage in tumor cells [48]. Consequently, combining redox-cycling quinones with radiotherapy represents an attractive strategy to enhance tumor-selective oxidative stress while minimizing damage to normal tissues [49].

**ATO**, which is currently undergoing clinical evaluation for cancer therapy, suppresses mitochondrial respiration, reduces hypoxia-associated signaling, and overcomes hypoxia-driven therapeutic resistance in several tumor types, including non-small cell lung cancer [50,51]. **Mito-ATO** derivatives are expected to further enhance these antitumor effects through a dual mechanism of action. First, they potently inhibit mitochondrial oxygen consumption, thereby improving tumor oxygenation and increasing radiosensitivity. Second, they undergo redox cycling to generate semiquinone intermediates that produce ROS, a process that is further amplified by ionizing radiation [52]. The increased superoxide production observed with **Mito-ATO** analogs, such as **MitoF-ATO**, together with their potent inhibition of mitochondrial respiration, supports this dual mechanism of radiosensitization.

Collectively, these findings identify mitochondria-targeted atovaquone derivatives as promising next-generation radiosensitizers that simultaneously alleviate tumor hypoxia and amplify radiation-induced oxidative stress, thereby providing a strong rationale for their further preclinical and clinical evaluation.

Beyond cancer therapeutics, **Mito-ATO** derivatives may also have therapeutic value in infectious diseases. Although **ATO** is a highly effective antimalarial drug, the emergence of *Plasmodium* strains resistant to Qo-site inhibition of mitochondrial complex III threatens its long-term clinical utility [[53], [54], [55]]. Our computational analyses predict that **Mito-ATO** derivatives (**MitoR-ATOs**) preferentially bind to the Qi site of complex III rather than the canonical Qo site. This distinct binding mode raises the possibility that these compounds could retain activity against parasites that have acquired resistance to conventional Qo-site inhibitors, providing a potential strategy to overcome an important mechanism of antimalarial drug resistance.

### 3.6 Study limitations

We and others have previously established rigorous analytical methods to identify the specific marker products formed when mitochondria-targeted fluorescent probes react with reactive oxygen and nitrogen species [56,57]. For example, superoxide reacts with hydroethidine (HE) to generate the diagnostic product 2-hydroxyethidium (2-OH-E^+^), whereas the mitochondria-targeted analog Mito-HE forms the corresponding product, Mito-2-OH-E^+^ [58,59].

A major limitation of these probes is that HE and Mito-HE can also be oxidized by heme proteins, hydrogen peroxide, and other cellular oxidants to produce nonspecific oxidation products (E^+^ and Mito-E^+^). Because the specific and nonspecific products exhibit nearly identical fluorescence spectra, conventional fluorescence microscopy and flow cytometry cannot distinguish superoxide-specific oxidation from nonspecific oxidation in intact cells [10].

Although liquid chromatography–mass spectrometry (LC–MS) methods enable selective quantification of the superoxide-specific product 2-OH-E^+^ in cellular extracts, analogous measurements for Mito-HE-derived products remain technically challenging because of inefficient extraction and the lack of suitable internal standards. The development of deuterated Mito-HE analogs and improved quantitative LC–MS methodologies should help overcome some of these limitations [57,60].

These technical constraints prevented direct quantification of superoxide generation by Mito-ATO and its analogs in intact cells. Future studies using isolated mitochondria or submitochondrial particles, where probe delivery and product recovery can be more tightly controlled, should facilitate quantitative assessment of substituent-dependent redox cycling and its relationship to mitochondrial complex III inhibition [61].

In addition to these analytical limitations, there are limitations with respect to in vivo validation of mitochondrial redox cycling of **Mito-ATOs**. Current mass spectrometry-based approaches for mitochondrial superoxide-specific probes are also limited in their ability to accurately determine the ratio of oxidized probe to the parent probe within living tissues, an important parameter for quantitative assessment of mitochondrial oxidant production [60].

Another in vivo restriction is the ready availability of a luciferase-transfected animal model for oxidation of pro-luciferin probes [62]. Another limitation is the restricted availability of suitable animal models for real-time in vivo imaging of mitochondrial oxidant production. Bioluminescence approaches based on oxidation of mitochondria-targeted pro-luciferin probes require luciferase-expressing animal models, which are not widely available and limit broader application of this methodology [62].

## 4 Conclusions

We synthesized a series of Mito-ATO derivatives and established an integrated experimental and computational platform to evaluate their redox properties, mitochondrial oxygen consumption, and antiproliferative effects. Although electronic modification of the TPP^+^ moiety significantly altered redox cycling and superoxide generation, these changes had little effect on mitochondrial respiration or cancer cell proliferation.

Computational modeling revealed that replacing the hydroxyl group of atovaquone disrupts binding at the Qo site of mitochondrial complex III while promoting interaction with the Qi site, suggesting an alternative mechanism of complex III inhibition. In contrast, elongation of the alkyl linker abolished complex III inhibition, underscoring the critical role of molecular structure in determining target engagement and biological activity.

Collectively, these findings demonstrate that modulation of the electronic properties of the TPP^+^ moiety alone is insufficient to predict the biological activity of mitochondria-targeted agents. Instead, optimal activity requires a balance between electronic properties and structural features that govern the ligand–protein interactions. These results provide an important framework for the development of next-generation mitochondria-targeted therapeutics with improved anticancer efficacy.

## CRediT authorship contribution statement

**Bruna Rafaela Pereira Resende:** Conceptualization, Data curation, Formal analysis, Investigation, Methodology, Software, Validation, Visualization, Writing – original draft, Writing – review & editing. **Gang Cheng:** Conceptualization, Data curation, Methodology, Software, Resources, Validation, Formal analysis, Project administration, Investigation, Visualization, Funding acquisition, Writing – review & editing. **Gabriel Canard:** Software, Investigation, Methodology, Data curation, Writing – review & editing. **Didier Siri:** Formal analysis, Writing – review & editing. **Vanessa Leone:** Formal analysis, Methodology, Writing – review & editing. **Micael Hardy:** Conceptualization, Data curation, Funding acquisition, Formal analysis, Methodology, Investigation, Resources, Project administration, Validation, Visualization, Writing – review & editing, Supervision, Software. **Balaraman Kalyanaraman:** Conceptualization, Data curation, Funding acquisition, Investigation, Project administration, Methodology, Resources, Supervision, Validation, Visualization, Writing – review & editing.

## Declaration of competing interests

Balaraman Kalyanaraman (BK) and Micael Hardy (MH) are inventors of US Patent No. 10,836,782/European Patent No. 3307254, “Mito-honokiol compounds and methods of synthesis and use thereof.” BK, MH, and Gang Cheng are inventors of US Patent No WO 2021081500, “Mitochondria-targeted atovaquone: a more potent and more effective antitumor, antimicrobial, and antimalarial drug.” The other authors have no competing interests to declare.
