# Supplementary material for: Redox and structural determinants of mitochondrial complex III inhibition by triphenylphosphonium-conjugated atovaquone analogs
Source: Redox Biol. 2026 Aug 28;97:104370. doi: 10.1016/j.redox.2026.104370 (PMC13571602; doi:10.1016/j.redox.2026.104370)
Supplement: Supplementary file 1 [file mmc1.docx]

Supplementary information

**Redox and structural determinants of mitochondrial complex III inhibition by triphenylphosphonium-conjugated atovaquone analogs**

Bruna Rafaela Pereira Resende,^a^ Gang Cheng,^b^ Gabriel Canard,^c^ Didier Siri,^a^ Vanessa Leone,^b^ Micael Hardy,^a,*^ Balaraman Kalyanaraman^b,*^

^a^Aix-Marseille Univ, CNRS, ICR, UMR 7273, Marseille, France
^b^Department of Biophysics, 8701 Watertown Plank Road, Milwaukee, WI, United States
^c^Aix-Marseille Univ, CNRS, CINaM, UMR 7325, Marseille, France

*Corresponding authors:

- Balaraman Kalyanaraman, Department of Biophysics, Medical College of Wisconsin, 8701 Watertown Plank Road, Milwaukee, WI 53226, United States; balarama@mcw.edu; 414-955-4000
- Micael Hardy, Aix-Marseille Univ, CNRS, ICR, UMR 7273, Marseille, France; micael.hardy@univ-amu.fr; 0665200299

# Table of contents

[Fig. S1. Chemical structures of novel MitoR-ATO analogs.	5](#_Toc235649936)

[Fig. S2. ^1^H NMR (400.13 MHz, CDCl_3_) spectra of ATO-Br.	6](#_Toc235649937)

[Fig. S3. ^1^H NMR (400.13 MHz, CDCl_3_) spectra of MitoCH_3_-ATO.	7](#_Toc235649938)

[Fig. S4. ^31^P NMR (161.98 MHz, CDCl_3_) spectra of MitoCH_3_-ATO.	7](#_Toc235649939)

[Fig. S5. ^13^C DEPTQ-135 NMR (100.58 MHz, CDCl_3_) spectra of MitoCH_3_-ATO.	8](#_Toc235649940)

[Fig. S6. ESI^+^ HR-MS spectra of MitoCH_3_-ATO [M]^+^ cation.	8](#_Toc235649941)

[Fig. S7. ^1^H NMR (400.13 MHz, CDCl_3_) spectra of MitoF-ATO.	9](#_Toc235649942)

[Fig. S8. ^19^F NMR (376.95 MHz, CDCl_3_) spectra of MitoF-ATO.	9](#_Toc235649943)

[Fig. S9. ^31^P NMR (161.98 MHz, CDCl_3_) spectra of MitoF-ATO.	10](#_Toc235649944)

[Fig. S10. ^13^C NMR (100.58 MHz, CDCl_3_) spectra of MitoF-ATO.	10](#_Toc235649945)

[Fig. S11. ESI^+^ HR-MS spectra of MitoF-ATO [M]+ cation.	11](#_Toc235649946)

[Fig. S12. ^1^H NMR (400.13 MHz, CDCl_3_) spectra of MitoCF_3_-ATO.	11](#_Toc235649947)

[Fig. S13. ^19^F NMR (376.95 MHz, CDCl_3_) spectra of MitoCF_3_-ATO.	12](#_Toc235649948)

[Fig. S14. ^31^P NMR (161.98 MHz, CDCl_3_) spectra of MitoCF_3_-ATO.	12](#_Toc235649949)

[Fig. S15. ^13^C NMR (100.58 MHz, CDCl_3_) spectra of MitoCF_3_-ATO.	13](#_Toc235649950)

[Fig. S16. ESI^+^ HR-MS spectra of MitoCF_3_-ATO [M]+ cation.	13](#_Toc235649951)

[Fig. S17. MitoCH_3_-ATO and its cyclic voltammogram.	14](#_Toc235649952)

[Fig. S18. MitoF-ATO and its voltammogram. The dotted line represents the reversibility studies for the selected electrochemical process.	15](#_Toc235649953)

[Fig. S19. MitoCF_3_-ATO and its voltammogram. The dotted line represents the reversibility studies for the selected electrochemical process.	15](#_Toc235649954)

[Fig. S20. EPR spectra obtained after incubation of a mixture containing reduced nicotinamide adenine dinucleotide (NADH, 0.2 mM), xanthine oxidase (XO, 25 mU.mL^-1^), DTPA (1 mM), and DIPPMPO (50 mM) in a phosphate buffer (0.05 M, pH 7.4). On the left, the spectra were recorded at a rate of 1 full scan/min up to 20 min (20 consecutive spectra). On the right, the spectrum is taken at 20 minutes of incubation.	16](#_Toc235649955)

[Fig. S21. As in Fig. S20, but in the presence of any MitoR-ATO (100 µM) and SOD (600 U.mL^-1^).	16](#_Toc235649956)

[Fig. S22. As in Fig. S20, but in the presence of ATO (100 µM).	16](#_Toc235649957)

[Fig. S23. As in Fig. S20, but in the presence of Mito-ATO (100 µM).	17](#_Toc235649958)

[Fig. S24. As in Fig. S20, but in the presence of Mito_16_-ATO (100 µM).	17](#_Toc235649959)

[Fig. S25. As in Fig. S20, but in the presence of MitoCH_3_-ATO (100 µM).	17](#_Toc235649960)

[Fig. S26. As in Fig. S20, but in the presence of MitoF-ATO (100 µM).	17](#_Toc235649961)

[Fig. S27. As in Fig. S20, but in the presence of MitoCF_3_-ATO (100 µM).	18](#_Toc235649962)

[Fig. S28. As in Fig. S20, but in the presence of Mito-ATO (50 µM).	18](#_Toc235649963)

[Fig. S29. As in Fig. S20, but in the presence of MitoCF_3_-ATO (50 µM).	18](#_Toc235649964)

[Fig. S30. As in Fig. S20, but in the presence of MitoCF_3_-ATO (25 µM).	18](#_Toc235649965)

[Fig. S31. Effect of the presence of ATO on the rate of NADH oxidation in a NADH/XO cell-free system. Data shown are the mean±SD, n=3.	19](#_Toc235649966)

[Fig. S32. As in Fig. S31, but in the presence of Mito-ATO.	19](#_Toc235649967)

[Fig. S33. As in Fig. S31, but in the presence of MitoCH_3_-ATO.	20](#_Toc235649968)

[Fig. S34. As in Fig. S31, but in the presence of MitoF-ATO.	20](#_Toc235649969)

[Fig. S35. As in Fig. S31, but in the presence of MitoCF_3_-ATO.	21](#_Toc235649970)

[Fig. S36. Full growth-inhibition curves for MitoF-ATO, MitoCH_3_-ATO, and MitoCF_3_-ATO on the proliferation of MiaPaCa-2 pancreatic cancer are shown. Cell proliferation is expressed as relative confluence, normalized to untreated control cells (100%). The IC_50_ values were calculated at the time point when control cells reached ~100% confluence. Data represent mean ± SD (n = 4).	21](#_Toc235649971)

[Fig. S37. Full growth-inhibition curves for MitoF-ATO, MitoCH_3_-ATO, and MitoCF_3_-ATO on the proliferation of MDA-MB-231 pancreatic cancer are shown. Cell proliferation is expressed as relative confluence, normalized to untreated control cells (100%). The IC_50_ values were calculated at the time point when control cells reached ~100% confluence. Data represent mean ± SD (n = 4).	22](#_Toc235649972)

[Fig. S38. Multiple sequence alignment of cytochrome *b* subunit using CLUSTAL O(1.2.4).[2] Species are *Bos Taurus* (UniProt ID: P00157), *Homo Sapiens* (UniProt ID: P00156), *Yarrowia lipolytica* (UniProt ID: Q9B6D0), and *Saccharomyces cerevisiae* (UniProt ID: P00163), respectively.	23](#_Toc235649973)

[Fig. S39. Multiple sequence alignment of cytochrome *b-c_1_* Rieske protein subunit using CLUSTAL O(1.2.4).[2] Species are *Homo Sapiens* (UniProt ID: P47985), *Bos Taurus* (UniProt ID: P13272), *Yarrowia lipolytica* (UniProt ID: Q6CI02), and *Saccharomyces cerevisiae* (UniProt ID: P08067), respectively.	23](#_Toc235649974)

[Fig. S40. Panel 1, view of the Q_o_ site of the X-ray structure of yeast cyt *bc_1_* (4PD4) with ATO bound (calculated pose in yellow, crystallographic pose in green). ATO calculated pose is part of a 93-pose cluster, displayed pose has a docking score of -11.09 kcal.mol^-1^. Panel 2, view of the Q_o_ site of the X-ray structure of bovine cyt *bc_1_* (1SQX) with ATO and SMA bound (ATO calculated pose in yellow, SMA crystallographic pose in green). ATO calculated pose is part of a 59-pose cluster, displayed pose has a docking score of -10.65 kcal.mol^-1^. Panel 3, view of the Q_o_ site of the X-ray structure of yeast cyt *bc_1_* (8AB7) with ATO bound (calculated pose in yellow, crystallographic pose in green). ATO calculated pose is part of a 93-pose cluster, displayed pose has a docking score of -11.84 kcal.mol^-1^	24](#_Toc235649975)

[Fig. S41. Panel 1, view of the Q_o_ site of the X-ray structure of yeast cyt *bc_1_* (3CX5) with SMA (Stigmatellin A) bound (calculated pose in blue, crystallographic pose in green). SMA calculated pose is part of a 41-pose cluster, displayed pose has a docking score of -10.42 kcal.mol^-1^. Panel 2, view of the Q_o_ site of the X-ray structure of yeast cyt *bc_1_* (4PD4) with ATO and SMA bound (ATO calculated pose in yellow, SMA calculated pose in blue). ATO calculated pose is part of a 93-pose cluster, displayed pose has a docking score of -11.09 kcal.mol^-1^. SMA calculated pose is part of a 14-pose cluster, displayed pose has a docking score of -9.99 kcal.mol^-1^.	24](#_Toc235649976)

[Fig. S42. Panel 1, view of the Q_o_ site of the X-ray structure of the ATO-sensitive yeast cyt *bc_1_* (4PD4) with ATO and SMA bound (ATO calculated pose in yellow, SMA calculated pose in blue). ATO calculated pose is part of a 93-pose cluster, displayed pose has a docking score of -11.09 kcal.mol^-1^. SMA calculated pose is part of a 14-pose cluster, displayed pose has a docking score of -9.99 kcal.mol^-1^. Panel B, view of the Q_o_ site of the X-ray structure of the ATO-resistant yeast cyt *bc_1_* (3CX5) with ATO and SMA bound (ATO calculated pose in yellow, SMA calculated pose in blue). ATO calculated pose is outside of the pocket. SMA calculated pose is part of a 41-pose cluster, displayed pose has a docking score of -10.42 kcal.mol^-1^.	25](#_Toc235649977)

[Fig. S43. ATO's fluctuation with respect to the MCIII Q_o_ site (PDB: 4PD4).	25](#_Toc235649978)

[Fig. S44. Protein RMSD (α-carbons aligned, blue) and ATO’s RMSD (red) over the 100 ns MD simulation.	26](#_Toc235649979)

[Fig. S45. Panel 1, docked ATO pose interactions with protein residues at least 30% of the time of the 100 ns MD simulation (hydrophobic residues are represented in green, polar residues in blue, negatively charged residues in orange; backbone H-bonds are represented by solid pink arrows, side-chain H-bonds by dashed pink arrows). Panel 2, Protein-ligand contacts per residue index over the 100 ns MD simulation.	26](#_Toc235649980)

[Fig. S46. Mito-ATO's fluctuation with respect to the MCIII Q_i_ site (PDB: 4PD4).	27](#_Toc235649981)

[Fig. S47. Protein RMSD (α-carbons aligned, blue) and Mito-ATO’s RMSD (red) over the 100 ns MD simulation.	27](#_Toc235649982)

[Fig. S48. Panel 1, docked Mito-ATO pose interactions with protein residues at least 30% of the time of the 100 ns MD simulation (positively charged residues are represented in blue, negatively charged residues in orange; backbone H-bonds are represented by solid pink arrows, side-chain H-bonds by dashed pink arrows). Panel 2, Protein-ligand contacts per residue index over the 100 ns MD simulation.	28](#_Toc235649983)

[Fig. S49. MitoCF_3_-ATO's fluctuation with respect to the MCIII Qi site (PDB: 4PD4).	28](#_Toc235649984)

[Fig. S50. Protein RMSD (α-carbons aligned, blue) and MitoCF_3_-ATO’s RMSD (red) over the 100 ns MD simulation.	29](#_Toc235649985)

[Fig. S51. Panel 1, docked MitoCF_3_-ATO pose interactions with protein residues at least 30% of the time of the 100 ns MD simulation (hydrophobic are represented in green, π-π stacking interactions are in green). Panel 2, Protein-ligand contacts per residue index over the 100 ns MD simulation.	29](#_Toc235649986)

[Fig. S52. MitoCH_3_-ATO's fluctuation with respect to the MCIII Qi site (PDB: 4PD4).	30](#_Toc235649987)

[Fig. S53. Protein RMSD (α-carbons aligned, blue) and MitoCH_3_-ATO’s RMSD (red) over the 100 ns MD simulation.	30](#_Toc235649988)

[Fig. S54. Panel 1, docked MitoCH_3_-ATO pose interactions with protein residues at least 30% of the time of the 100 ns MD simulation (hydrophobic residues are represented in green, polar residues in blue, π-π stacking interactions are in green). Panel 2, Protein-ligand contacts per residue index over the 100 ns MD simulation.	31](#_Toc235649989)

[Fig. S55. MitoF-ATO's fluctuation with respect to the MCIII Qi site (PDB: 4PD4).	31](#_Toc235649990)

[Fig. S56. Protein RMSD (α-carbons aligned, blue) and MitoF-ATO’s RMSD (red) over the 100 ns MD simulation.	32](#_Toc235649991)

[Fig. S57. Panel 1, docked MitoF-ATO pose interactions with protein residues at least 30% of the time of the 100 ns MD simulation (hydrophobic residues are represented in green). Panel 2, Protein-ligand contacts per residue index over the 100 ns MD simulation.	32](#_Toc235649992)

[Fig. S58. Mito_16_-ATO's fluctuation with respect to the MCIII Qi site (PDB: 4PD4).	33](#_Toc235649993)

[Fig. S59. Protein RMSD (α-carbons aligned, blue) and Mito_16_-ATO’s RMSD (red) over the 100 ns MD simulation.	33](#_Toc235649994)

[Fig. S60. Panel 1, docked Mito_16_-ATO pose interactions with protein residues at least 30% of the time of the 100 ns MD simulation (hydrophobic residues are represented in green). Panel 2, Protein-ligand contacts per residue index over the 100 ns MD simulation.	34](#_Toc235649995)

# Chemistry

## 1. Chemical structures synthesized

Fig. S1. Chemical structures of novel MitoR-ATO analogs.

## 2. NMR Spectra

### 2.1. ATO-Br

Fig. S2. ^1^H NMR (400.13 MHz, CDCl_3_) spectra of ATO-Br.

### 2.2. MitoCH_3_-ATO

Fig. S3. ^1^H NMR (400.13 MHz, CDCl_3_) spectra of MitoCH_3_-ATO.

Fig. S4. ^31^P NMR (161.98 MHz, CDCl_3_) spectra of MitoCH_3_-ATO.

Fig. S5. ^13^C DEPTQ-135 NMR (100.58 MHz, CDCl_3_) spectra of MitoCH_3_-ATO.

Fig. S6. ESI^+^ HR-MS spectra of MitoCH_3_-ATO [M]^+^ cation.

### 2.3. MitoF-ATO

Fig. S7. ^1^H NMR (400.13 MHz, CDCl_3_) spectra of MitoF-ATO.

Fig. S8. ^19^F NMR (376.95 MHz, CDCl_3_) spectra of MitoF-ATO.

Fig. S9. ^31^P NMR (161.98 MHz, CDCl_3_) spectra of MitoF-ATO.

Fig. S10. ^13^C NMR (100.58 MHz, CDCl_3_) spectra of MitoF-ATO.

Fig. S11. ESI^+^ HR-MS spectra of MitoF-ATO [M]+ cation.

### 2.4. MitoCF_3_-ATO

Fig. S12. ^1^H NMR (400.13 MHz, CDCl_3_) spectra of MitoCF_3_-ATO.

Fig. S13. ^19^F NMR (376.95 MHz, CDCl_3_) spectra of MitoCF_3_-ATO.

Fig. S14. ^31^P NMR (161.98 MHz, CDCl_3_) spectra of MitoCF_3_-ATO.

Fig. S15. ^13^C NMR (100.58 MHz, CDCl_3_) spectra of MitoCF_3_-ATO.

Fig. S16. ESI^+^ HR-MS spectra of MitoCF_3_-ATO [M]+ cation.

# Calculated values of the octanol/water partition coefficients

Table S1. Calculated values of the octanol/water partition coefficients.

# Cyclic Voltammetry

## 1. MitoCH_3_-ATO

|  |  |
| --- | --- |

Fig. S17. MitoCH_3_-ATO and its cyclic voltammogram.

Table S2. Redox parameters of MitoCH_3_-ATO.

|  | E_1/2_ red_2_ | Ep_c2_ | E_1/2_ red_1_ | Ep_c1_ | Ep_a1_ |
| --- | --- | --- | --- | --- | --- |
| **MitoCH_3_-ATO** | - | - | -0.83 **1e** | -0.86 | -0.79 |

## 2. MitoF-ATO

|  |  |
| --- | --- |

Fig. S18. MitoF-ATO and its voltammogram. The dotted line represents the reversibility studies for the selected electrochemical process.

Table S3. Redox parameters of MitoF-ATO.

|  | E_1/2_ red_2_ | Ep_c2_ | E_1/2_ red_1_ | Ep_c1_ | Ep_a1_ |
| --- | --- | --- | --- | --- | --- |
| **MitoF-ATO** | -1.30 **1e** | -1.36 | -0.82 **1e** | -0.85 | -0.77 |

## 3. MitoCF_3_-ATO

|  |  |
| --- | --- |

Fig. S19. MitoCF_3_-ATO and its voltammogram. The dotted line represents the reversibility studies for the selected electrochemical process.

Table S4. Redox parameters of MitoCF_3_-ATO.

|  | E_1/2_ red_2_ | Ep_c2_ | E_1/2_ red_1_ | Ep_c1_ | Ep_a1_ |
| --- | --- | --- | --- | --- | --- |
| **MitoCF_3_-ATO** | -1.26 **1e** | -1.34 | -0.82 **1e** | -0.86 | -0.76 |

# Redox-cycling activity

## 1. EPR coupled to the spin-trapping technique

Table S5. EPR parameters of DIPPMPO-OOH superoxide adduct.

| **Adduct** | **Redox System** | **Diastereoisomer** | **Conformer** | **k(s^-1^)** | **A_P_/mT** | **A_N_/mT** | **A_Hβ_/mT** |
| --- | --- | --- | --- | --- | --- | --- | --- |
| DIPPMPO-OOH | NADH/XO  pH 7.4 | *trans* 90 % | T1 (57%) | 0.2 | 4.93 | 1.32 | 1.02 |
|  |  |  | T2 (43%) | ×10^8^ | 5.10 | 1.29 | 1.23 |
|  |  | *cis* 10% |  |  | 4.20 | 1.38 | 0.90 |

### 1.1. Control: No MitoR-ATO

Fig. S20. EPR spectra obtained after incubation of a mixture containing reduced nicotinamide adenine dinucleotide (NADH, 0.2 mM), xanthine oxidase (XO, 25 mU.mL^-1^), DTPA (1 mM), and DIPPMPO (50 mM) in a phosphate buffer (0.05 M, pH 7.4). On the left, the spectra were recorded at a rate of 1 full scan/min up to 20 min (20 consecutive spectra). On the right, the spectrum is taken at 20 minutes of incubation.

### 1.2. Control: Any MitoR-ATO + SOD

Fig. S21. As in Fig. S20, but in the presence of any MitoR-ATO (100 µM) and SOD (600 U.mL^-1^).

### 1.3. Control: ATO

Fig. S22. As in Fig. S20, but in the presence of ATO (100 µM).

### 1.4. Control: Mito-ATO (100 µM)

Described as a redox-cycling agent.[1]

Fig. S23. As in Fig. S20, but in the presence of Mito-ATO (100 µM).

### 1.5. Control: Mito_16_-ATO

Described as being unable to redox-cycle.[1]

Fig. S24. As in Fig. S20, but in the presence of Mito_16_-ATO (100 µM).

### 1.6. MitoCH_3_-ATO

Fig. S25. As in Fig. S20, but in the presence of MitoCH_3_-ATO (100 µM).

### 1.7. MitoF-ATO

Fig. S26. As in Fig. S20, but in the presence of MitoF-ATO (100 µM).

### 1.8. MitoCF_3_-ATO (100 µM)

Fig. S27. As in Fig. S20, but in the presence of MitoCF_3_-ATO (100 µM).

### 1.9. Mito-ATO (50 µM)

Fig. S28. As in Fig. S20, but in the presence of Mito-ATO (50 µM).

### 1.10. MitoCF_3_-ATO (50 µM)

Fig. S29. As in Fig. S20, but in the presence of MitoCF_3_-ATO (50 µM).

### 1.11. MitoCF_3_-ATO (25 µM)

Fig. S30. As in Fig. S20, but in the presence of MitoCF_3_-ATO (25 µM).

## 2. NADH oxidation kinetics

### 2.1. ATO

Fig. S31. Effect of the presence of ATO on the rate of NADH oxidation in a NADH/XO cell-free system. Data shown are the mean±SD, n=3.

### 2.2. Mito-ATO

Fig. S32. As in Fig. S31, but in the presence of Mito-ATO.

### 2.3. MitoCH_3_-ATO

Fig. S33. As in Fig. S31, but in the presence of MitoCH_3_-ATO.

### 2.4. MitoF-ATO

Fig. S34. As in Fig. S31, but in the presence of MitoF-ATO.

### 2.5. MitoCF_3_-ATO

Fig. S35. As in Fig. S31, but in the presence of MitoCF_3_-ATO.

# Cell proliferation data

## 1. On MiaPaCa-2 cells

Fig. S36. Full growth-inhibition curves for MitoF-ATO, MitoCH_3_-ATO, and MitoCF_3_-ATO on the proliferation of MiaPaCa-2 pancreatic cancer are shown. Cell proliferation is expressed as relative confluence, normalized to untreated control cells (100%). The IC_50_ values were calculated at the time point when control cells reached ~100% confluence. Data represent mean ± SD (n = 4).

## 2. On MDA-MB-231 cells

Fig. S37. Full growth-inhibition curves for MitoF-ATO, MitoCH_3_-ATO, and MitoCF_3_-ATO on the proliferation of MDA-MB-231 pancreatic cancer are shown. Cell proliferation is expressed as relative confluence, normalized to untreated control cells (100%). The IC_50_ values were calculated at the time point when control cells reached ~100% confluence. Data represent mean ± SD (n = 4).

# Computational studies

## 1. Multi-sequence alignment

Fig. S38. Multiple sequence alignment of cytochrome *b* subunit using CLUSTAL O(1.2.4).[2] Species are *Bos Taurus* (UniProt ID: P00157), *Homo Sapiens* (UniProt ID: P00156), *Yarrowia lipolytica* (UniProt ID: Q9B6D0), and *Saccharomyces cerevisiae* (UniProt ID: P00163), respectively.

Fig. S39. Multiple sequence alignment of cytochrome *b-c_1_* Rieske protein subunit using CLUSTAL O(1.2.4).[2] Species are *Homo Sapiens* (UniProt ID: P47985), *Bos Taurus* (UniProt ID: P13272), *Yarrowia lipolytica* (UniProt ID: Q6CI02), and *Saccharomyces cerevisiae* (UniProt ID: P08067), respectively.

## 2. Docking ATO on different species’ MCIII Q_o_ site

Fig. S40. Panel 1, view of the Q_o_ site of the X-ray structure of yeast cyt *bc_1_* (4PD4) with ATO bound (calculated pose in yellow, crystallographic pose in green). ATO calculated pose is part of a 93-pose cluster, displayed pose has a docking score of -11.09 kcal.mol^-1^. Panel 2, view of the Q_o_ site of the X-ray structure of bovine cyt *bc_1_* (1SQX) with ATO and SMA bound (ATO calculated pose in yellow, SMA crystallographic pose in green). ATO calculated pose is part of a 59-pose cluster, displayed pose has a docking score of -10.65 kcal.mol^-1^. Panel 3, view of the Q_o_ site of the X-ray structure of yeast cyt *bc_1_* (8AB7) with ATO bound (calculated pose in yellow, crystallographic pose in green). ATO calculated pose is part of a 93-pose cluster, displayed pose has a docking score of -11.84 kcal.mol^-1^

## 3. Docking SMA on MCIII’s Q_o_ site

Fig. S41. Panel 1, view of the Q_o_ site of the X-ray structure of yeast cyt *bc_1_* (3CX5) with SMA (Stigmatellin A) bound (calculated pose in blue, crystallographic pose in green). SMA calculated pose is part of a 41-pose cluster, displayed pose has a docking score of -10.42 kcal.mol^-1^. Panel 2, view of the Q_o_ site of the X-ray structure of yeast cyt *bc_1_* (4PD4) with ATO and SMA bound (ATO calculated pose in yellow, SMA calculated pose in blue). ATO calculated pose is part of a 93-pose cluster, displayed pose has a docking score of -11.09 kcal.mol^-1^. SMA calculated pose is part of a 14-pose cluster, displayed pose has a docking score of -9.99 kcal.mol^-1^.

## 4. Docking ATO and SMA on ATO-sensitive and resistant Q_o_ site

Fig. S42. Panel 1, view of the Q_o_ site of the X-ray structure of the ATO-sensitive yeast cyt *bc_1_* (4PD4) with ATO and SMA bound (ATO calculated pose in yellow, SMA calculated pose in blue). ATO calculated pose is part of a 93-pose cluster, displayed pose has a docking score of -11.09 kcal.mol^-1^. SMA calculated pose is part of a 14-pose cluster, displayed pose has a docking score of -9.99 kcal.mol^-1^. Panel B, view of the Q_o_ site of the X-ray structure of the ATO-resistant yeast cyt *bc_1_* (3CX5) with ATO and SMA bound (ATO calculated pose in yellow, SMA calculated pose in blue). ATO calculated pose is outside of the pocket. SMA calculated pose is part of a 41-pose cluster, displayed pose has a docking score of -10.42 kcal.mol^-1^.

## 5. ATO in Q_o_: Ligand RMSF, protein and ligand RMSD, and interactions panel

### 5.1. Ligand RMSF

Fig. S43. ATO's fluctuation with respect to the MCIII Q_o_ site (PDB: 4PD4).

### 5.2. Protein and Ligand RMSD

Fig. S44. Protein RMSD (α-carbons aligned, blue) and ATO’s RMSD (red) over the 100 ns MD simulation.

### 5.3. Interactions panel

Fig. S45. Panel 1, docked ATO pose interactions with protein residues at least 30% of the time of the 100 ns MD simulation (hydrophobic residues are represented in green, polar residues in blue, negatively charged residues in orange; backbone H-bonds are represented by solid pink arrows, side-chain H-bonds by dashed pink arrows). Panel 2, Protein-ligand contacts per residue index over the 100 ns MD simulation.

## 6. Mito-ATO in Q_i_: Ligand RMSF, protein and ligand RMSD, and interactions panel

### 6.1. Ligand RMSF

Fig. S46. Mito-ATO's fluctuation with respect to the MCIII Q_i_ site (PDB: 4PD4).

### 6.2. Protein and Ligand RMSD

Fig. S47. Protein RMSD (α-carbons aligned, blue) and Mito-ATO’s RMSD (red) over the 100 ns MD simulation.

### 6.3. Interactions panel

Fig. S48. Panel 1, docked Mito-ATO pose interactions with protein residues at least 30% of the time of the 100 ns MD simulation (positively charged residues are represented in blue, negatively charged residues in orange; backbone H-bonds are represented by solid pink arrows, side-chain H-bonds by dashed pink arrows). Panel 2, Protein-ligand contacts per residue index over the 100 ns MD simulation.

## 7. MitoCF_3_-ATO in Q_i_: Ligand RMSF, protein and ligand RMSD, and interactions panel

### 7.1. Ligand RMSF

Fig. S49. MitoCF_3_-ATO's fluctuation with respect to the MCIII Qi site (PDB: 4PD4).

### 7.2. Protein and Ligand RMSD

Fig. S50. Protein RMSD (α-carbons aligned, blue) and MitoCF_3_-ATO’s RMSD (red) over the 100 ns MD simulation.

### 7.3. Interactions panel

Fig. S51. Panel 1, docked MitoCF_3_-ATO pose interactions with protein residues at least 30% of the time of the 100 ns MD simulation (hydrophobic are represented in green, π-π stacking interactions are in green). Panel 2, Protein-ligand contacts per residue index over the 100 ns MD simulation.

## 8. MitoCH_3_-ATO in Q_i_: Ligand RMSF, protein and ligand RMSD, and interactions panel

### 8.1. Ligand RMSF

Fig. S52. MitoCH_3_-ATO's fluctuation with respect to the MCIII Qi site (PDB: 4PD4).

### 8.2. Protein and Ligand RMSD

Fig. S53. Protein RMSD (α-carbons aligned, blue) and MitoCH_3_-ATO’s RMSD (red) over the 100 ns MD simulation.

### 8.3. Interactions panel

Fig. S54. Panel 1, docked MitoCH_3_-ATO pose interactions with protein residues at least 30% of the time of the 100 ns MD simulation (hydrophobic residues are represented in green, polar residues in blue, π-π stacking interactions are in green). Panel 2, Protein-ligand contacts per residue index over the 100 ns MD simulation.

## 9. MitoF-ATO in Q_i_: Ligand RMSF, protein and ligand RMSD, and interactions panel

### 9.1. Ligand RMSF

Fig. S55. MitoF-ATO's fluctuation with respect to the MCIII Qi site (PDB: 4PD4).

### 9.2. Protein and Ligand RMSD

Fig. S56. Protein RMSD (α-carbons aligned, blue) and MitoF-ATO’s RMSD (red) over the 100 ns MD simulation.

### 9.3. Interactions panel

Fig. S57. Panel 1, docked MitoF-ATO pose interactions with protein residues at least 30% of the time of the 100 ns MD simulation (hydrophobic residues are represented in green). Panel 2, Protein-ligand contacts per residue index over the 100 ns MD simulation.

## 10. Mito_16_-ATO in Q_i_: Ligand RMSF, protein and ligand RMSD, and interactions panel

### 10.1. Ligand RMSF

Fig. S58. Mito_16_-ATO's fluctuation with respect to the MCIII Qi site (PDB: 4PD4).

### 10.2. Protein and Ligand RMSD

Fig. S59. Protein RMSD (α-carbons aligned, blue) and Mito_16_-ATO’s RMSD (red) over the 100 ns MD simulation.

### 10.3. Interactions panel

Fig. S60. Panel 1, docked Mito_16_-ATO pose interactions with protein residues at least 30% of the time of the 100 ns MD simulation (hydrophobic residues are represented in green). Panel 2, Protein-ligand contacts per residue index over the 100 ns MD simulation.

# Structure Activity Relationship

Table S6. Structure–activity relationship (SAR) of synthesized MitoR-ATO derivatives. n.d., not determined, (+), positive and (-), negative.

# References

[1]	G. Cheng, M. Hardy, P. Topchyan, R. Zander, P. Volberding, W. Cui, B. Kalyanaraman, Potent inhibition of tumour cell proliferation and immunoregulatory function by mitochondria-targeted atovaquone, Sci. Rep. 10 (2020) 17872.

[2]	F. Sievers, A. Wilm, D. Dineen, T.J. Gibson, K. Karplus, W. Li, R. Lopez, H. McWilliam, M. Remmert, J. Söding, J.D. Thompson, D.G. Higgins, Fast, scalable generation of high‐quality protein multiple sequence alignments using Clustal Omega, Mol. Syst. Biol. (2011).
